# Chemical Derivatization of Commercially Available Condensed and Hydrolyzable Tannins

**DOI:** 10.1021/acssuschemeng.1c02114

**Published:** 2021-07-16

**Authors:** Lili Zhen, Heiko Lange, Luc Zongo, Claudia Crestini

**Affiliations:** † University of Rome “Tor Vergata”, Department of Chemical Science and Technologies, Via della Ricerca Scientifica, 00133 Rome, Italy; ‡ CSGI—Center for Colloid and Surface Science, Via della Lastruccia 3, 50019 Sesto Fiorentino, Italy; § Department of Earth and Environmental Sciences, University of Milan-Bicocca, Piazza della Scienza 1, 20126 Milano, Italy; ∥ University of Venice “Ca” Foscari’, Department of Molecular Science and Nanosystems, Via Torino 155, 30170 Venice Mestre, Italy

**Keywords:** natural polyphenols, tannins, functional materials, copolymers, charged polymers

## Abstract

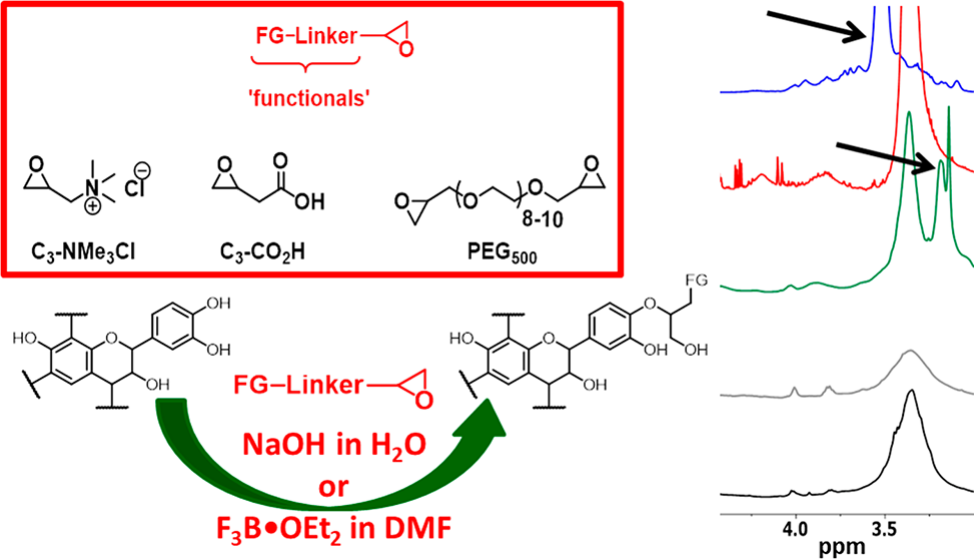

Novel valorization routes for tannins were opened by the development of a simple, straightforward, robust, and flexible approach to the selective functionalization of condensed and hydrolyzable tannins. Irrespective of the different degrees of polymerization, different commercial tannins were efficiently functionalized by the generation of an ether linkage bound to a short linker carrying the desired functional group. Functionalizations could be realized at varying degrees of technical loadings, i.e., amounts of introduced tannin-alien functionalities per number of phenolic hydroxyl groups. The same strategy was found suitable for the synthesis of polyethylene glycol-functionalized tannin copolymers. Condensed tannins functionalized with carboxylic acid moieties could be converted into a tannin–oligopeptide hybrid.

## Abstract 2

Commercially available condensed and hydrolyzable tannins were functionalized with different functional groups at different concentrations.

## Introduction

Eco-friendly chemical compounds in the form of plant extracts such as polyphenolic lignins and tannins are of utmost interest with respect to both industrial and biomedical applications.^1−4^ Especially, tannins represent one of the most versatile compendiums of polyphenolic compounds derived from biomass.^4,5^ Although not as abundant as lignin, they are much more widely used in everyday life due to their relatively easy isolation and traditionally well-known, albeit not always scientifically fully elucidated/understood, functional features.^4^ From a chemical and biological point of view, tannins are interesting because of the possibilities that arise in terms of the use and manipulation of features combined in a single structure.^3−7^ Numerous studies have demonstrated that tannins have many health benefits such as antioxidant,^8^ and anti-inflammatory properties,^9−11^ anti-mutagenic, and anti-carcinogenic activities,^9^ prevention and delay of cardiovascular diseases, and increase the lifespan and retard the onset of age-related markers.^8,12,13^ These findings scientifically sustain and illustrate the long-known beneficial effects of diets containing tannin-rich beverages and foods, such as green tea, fruits, and vegetables.

Chemically, tannins are interesting due to their metal-complexing capacities, their antioxidant character, and their capacities to undergo hydrophobic interactions either with other polyphenolic structures, e.g., especially tannins and lignins or with other functional biomacromolecules, e.g., proteins.^14^ The first type of interaction was recently used to form novel types of tannin micro- and nanocapsules and emulsions; when combined with the metal-complexing features, ultrasonication approaches yielded versatile systems suitable for targeted delivery and/or controlled release of actives.^15−22^

Tannins have been used as starting materials for the development of functional materials, including chemical modifications of the tannin core. Older patents describe tannins containing chemically introduced nitrogen functionalities as nature-derived polymeric coagulants in water and wastewater treatment operations.^23^ Hydrogels or wood preservatives have been reported on the basis of tannins modified with polyethylene glycols.^24,25^ Other cross-linking methods have been applied for the generation of more or less rigid tannin-based polymers and resins.^26,27^ A recent review has critically discussed these and other chemical modifications of tannins, including methods that change the core tannin structure itself.^28^

In an effort aiming at identifying novel valorization routes of tannins especially in the form of surface-modifying compounds for applications in antibiofilm formulations^29−36^ and as actives for functional textiles,^37−42^ a facile and scalable low-cost derivatization of commercially available tannins became necessary. The aim was to significantly broaden their actual scope of applicability without a concomitant complete loss of typical “tannin features” such as antioxidant or protein-complexing activities.

This paper describes the chemical derivatization of exemplary tannins using readily available epoxides. The starting tannins represent commercially available samples of higher quality grades; structural features of the tannins claimed by the suppliers have been validated prior to their use, as outlined in detail in previous publications,^43,44^ relying especially on NMR-based quantification of the OH groups for this work.^45−47^

## Experimental Section

### General Information

Reagents and solvents were purchased and used without further purification, unless stated otherwise, from Sigma-Aldrich and Carlo Erba. Tannins were purchased from various vendors as listed in Table 1 and used without further purification. 2-Oxiranylacetic acid was synthesized using published procedures.^48^*N*,*N*-Dimethylformamide (DMF) was dried according to a published protocol^49^ and stored over 4 Å molecular sieves.

**Table 1:** Details of Commercially Available Tannins Used in This Study

samples (species)	tannin type	supplier	code
OmniVin WG (*Vitis vinifera*)	condensed	OmniChem	***Vv***
OmniVin 20R (*Vitis vinifera*)	condensed	OmniChem	***Vv*-20**
MIMOSA ATO ME (*Acacia mearnsii*)	condensed	Figli di Guido Lapi	***Am***
Quebracho wood extract (*Schinopsis balansae*)	condensed	SilvaChimica	***Sb*W**
Tanal 01 (unknown)	hydrolyzable (gallotannin)	OmniChem	**Ta-01**
Tanal 04 (unknown)	hydrolyzable	OmniChem	**Ta-04**

### Functionalization of Tannins in Aqueous Media

#### Standard Procedure

Typically, 300 mg of tannin was dispersed in 1.8 mL of water, before a volume of 0.1 N aqueous sodium hydroxide was added that in terms of number of hydroxyl ions to 1.0 equiv of the total phenolic hydroxyl groups present in the tannin under the study, as determined by quantitative ^31^P NMR spectroscopy. The overall reaction volume was subsequently adjusted to 5 mL. After 1 h of stirring at approx. 50 °C, the epoxy-terminated functional, e.g., epoxide-terminated glycidyltrimethylammonium chloride (**C**_**3**_**-NMe**_**3**_**Cl**), eventually dissolved in a small amount of distilled water, was added dropwise over a time-span of 5 min in concentrations depending on the desired final technical loading; acid functionality (**C**_**3**_**-CO**_**2**_**H)** was added in form of its sodium salt in aqueous solution. The reaction mixture was stirred at approx. 50 °C for 4 h. The reaction was stopped by adjusting the pH to 3–4 using 1 N aqueous HCl. Subsequent isolation of the functionalized tannin was achieved following one of the general protocols described below.

### Functionalization of Tannins in Dry DMF

Typically, 300 mg of tannin was dispersed in 3 mL of dry DMF. The epoxy-terminated functional, e.g., epoxide-terminated glycidyltrimethylammonium chloride (**C**_**3**_**-NMe**_**3**_**Cl)**, eventually dissolved in a small amount of dry DMF, was added in concentrations depending on the desired final technical loading, typically in the range from 0.1 to 0.5 equiv to tannin phenolic OH groups. Boron trifluoride diethyl etherate (**F**_**3**_**B·OEt**_**2**_) (12 μL (2.5%)) as the catalyst was injected. The reaction mixture was stirred at approx. 50 °C for 4 h. Subsequent isolation of the functionalized tannin was achieved following one of the general protocols described below.

### Derivatization of Functionalized Tannin with an Oligopeptide

Typically, 20 mg of ***Sb*****W** functionalized with 2-oxiranylacetic acid (0.74 mmol carboxylic acid, 1.0 equiv), i.e., ***Sb*****W AcC**_**3**_ was weighed together with *N*,*N*-dimethylaminopyridine (**DMAP**) (0.11 mmol, 13.6 mg, 2.0 equiv) and 1-hydroxybenzotriazol (**HOBt**) (0.083 mmol, 11.3 mg, 1.5 equiv) in a small glass vial and dissolved in 800 μL of dry DMF. 1-Ethyl-3-(3-dimethylaminopropyl)carbodiimide (**EDC**) (0.055 mmol, 9.5 mg, 1.1 equiv) was dissolved in 200 μL of dry DMF and added to the reaction mixture at 0 °C. The system was stirred for 1 h before adding 1.0 equiv of the oligopeptide cholecystokinin fragment 30–33 (**Cfrag3033**). After stirring overnight, 2 mL of distilled H_2_O was added to stop the reaction. Isolation of the functionalized tannin was achieved following one of the general protocols described below.

### Isolation of Chemically Modified Tannins

#### Isolation by Means of Adsorption–Desorption Protocols

Reaction solutions were diluted 5 times with 20% (v/v) aqueous DMF and transferred to an Erlenmeyer flask and 200 mg of activated XAD resin was added. The flask was shaken for 8 h. After absorption, the resin was washed three times with an equal volume of distilled water to remove the DMF. For desorption, the tannin-containing XAD resin was separated from the aqueous phase and eluted with 60% (v/v) aqueous ethanol, typically using 40 mL portions, until the absorbance value at λ = 280 nm of the collected extracts, determined via ultraviolet–visible (UV–vis) spectroscopy, indicated complete desorption. Aqueous ethanol fractions were combined and concentrated using a rotary evaporator. The remaining traces of water were removed using a lyophilizer.

#### Isolation by Means of Dialysis Protocols

Quenched reaction solutions were transferred into a dialysis tube with a molecular weight cut-off (MWCO) of 500–1000 Da. Filled tubes were submerged in an amount of distilled water equal to 10 times the reaction volume for 3 days, under gentle stirring, with the water being replaced every 24 h. The dialyzed material was isolated by concentrating the aqueous solution using a rotary evaporator and freeze-drying the residue.

#### Isolation by Means of Precipitation

Functionalized tannins were precipitated at pH 2 by adding suitable volumes of 2 N aqueous HCl and were subsequently isolated by centrifugation (15 min, 5000 rpm). The initial pellet was resuspended in acidified water (pH 2) and subsequently reisolated. This washing of the pellet was repeated and the final pellet was freeze-dried for analysis.

### Nuclear Magnetic Resonance (NMR) Measurements

#### ^1^H NMR Measurements

An accurately weighed amount of analyte (about 10.0 mg) was dissolved in 600 μL of deuterated dimethyl sulfoxide (DMSO-*d*_6_). The mixture was transferred into 5 mm NMR tubes. Phthalimide (20 μL, 10 mg/mL in DMSO-*d*_6_) was added as an internal standard. The spectra were acquired on a Bruker 400 MHz spectrometer using the standard Bruker zg sequence (64 scans at 20 °C). NMR data were processed using MestreNova (Version 8.1.1, Mestrelab Research).

#### ^31^P NMR Measurements

The previously described procedure was followed.^43,45−47^ In brief, approx. 15 mg of tannin was accurately weighted and added to 450 μL of a mixture of pyridine/CDCl_3_ (1.6:1). One hundred microliters of the standard solution, prepared using *N*-hydroxy-5-norbornene-2,3-dicarboxylic acid imide (***e*****-HNDI**) at a concentration of 0.1 M in the abovementioned solvent mixture mixed with 50 mg/mL of Cr(III) acetylacetonate as the relaxation agent, was added, followed by 50 μL of 2-chloro-4,4,5,5-tetramethyl-1,3,2-dioxaphospholane (**Cl-TMPD**). After 1 h stirring at room temperature, the functionalized mixture was quantitatively transferred to a standard NMR tube for analysis. ^31^P NMR spectra were recorded on a Bruker 400 MHz spectrometer at 20 °C using an inverse gated decoupling sequence with a delay of 10 s between successive pulses. Chemical shifts were expressed in parts per million from 85% H_3_PO_4_ as an external reference. All chemical shifts reported are relative to the peak of the reaction product of water with **Cl-TMDP** at 132.2 ppm under the used conditions. NMR data were processed using MestreNova (Version 8.1.1, Mestrelab Research).

#### ^1^H-^13^C HSQC Measurements

Samples of around 50 mg were dissolved in 600 μL of DMSO-*d*_6_ (providing NMR sample solutions with concentrations of around 83 mg/mL); chromium(III) acetylacetonate was added as a spin-relaxing agent at a final concentration of ca. 1.5–1.75 mg/mL. HSQC spectra were recorded at 27 °C on a Bruker 700 MHz instrument equipped with TopSpin 2.1 software. Spectra were referenced to the residual signals of DMSO-*d*_6_ (2.49 ppm for ^1^H and 39.5 ppm for ^13^C spectra). ^1^H-^13^C HSQC spectra were obtained using 32 scans, employing the standard Bruker pulse program (hsqcegtpsisp2) with the following parameters for acquisition: TD = 2048 (F2), 512 (F1); SW = 13.0327 ppm (F2), 160 ppm (F1); O1 = 4200.54 Hz; O2 = 14083.02 Hz; D1 = 2 s; CNST2 = 145; acquisition time F2 channel = 112.34 ms; F1 channel = 8.7102 ms and the following parameters for processing: SI = 1024 (F2, F1), WDW = QSINE, LB = 1.00 Hz(F2), 0.30 Hz (F1); PH_mod = pk; baseline correction ABSG = 5 (F2, F1), BCFW = 1.00 ppm, BC_mod = quad (F2), no (F1); linear prediction = no (F2), LPfr (F1). Integration ranges as previously reported were applied. NMR data were processed using MestreNova (Version 8.1.1, Mestrelab Research).

### Matrix-Assisted Laser Desorption/Ionization–Time-of-flight Mass Spectrometry

MALDI-ToF analyses were performed using a Voyager-DE PRO Biospectrometry Workstation operated using Voyager operating software (version X). Samples were dissolved in water/acetone (4 mg/mL, 50/50 vol), and the solutions were mixed with the 2,6-dihydroxy-benzoic acid (**2,6-DHB**) matrix solution (10 mg/mL in acetone). For positive charged and non-ionic analytes, sodium chloride (NaCl) was added to the 2,6-dihydroxy-benzoic acid (**2,6-DHB**) solution (10 mg/mL in distilled water) to enhance ion formation. The sample and the matrix solutions were mixed as follows: 3 parts of the matrix solution, 3 parts of the sample solution, and 1 part NaCl solution; approx. 2.5 μL of the resulting mixture was placed on the MALDI sample holder. After drying overnight, the samples were analyzed using settings specifically optimized for each sample type.

### Gel Permeation Chromatography

Approx. 1 mg of natural or derivatized tannin was dissolved in 1 mL of DMSO containing 0.1% lithium chloride. A Shimadzu instrument was used consisting of a controller unit (CBM-20A), a pumping unit (LC 20AT), a degasser (DGU-20A3), a column oven (CTO-20AC), a diode array detector (SPD-M20A), and a refractive index detector (RID-10A)); the system was controlled using Shimadzu LabSolutions (Version 5.42 SP3). Three analytical GPC columns (each 7.5 × 30 mm^2^) in series were used for analysis: Agilent PLgel 5 μm 10 000 Å, followed by Agilent PLgel 5 μm 1000 Å, followed by Agilent PLgel 5 μm 500 Å. HPLC-grade DMSO (Chromasolv, Sigma-Aldrich) was used as the eluent at 70 °C column temperature. The run time at 0.25 mL min^–1^ flow rate was 20 min. Molecular weights were calculated from a linear calibration constructed with poly(styrene sulfonic acid) polymers (MW 4300–2.6 ×10^6^ g mol^–1^); analyses were run in duplicate.

## Results and Discussion

Different types of tannins representing various tannin classes were chosen for functionalization on the basis of their physicochemical characteristics, their high amounts of phenolic OH groups, and low contents in aliphatic OH groups suitable for derivatization and for their reported activities in antibiofilm-related applications.^30,50,51^ Characterization and thus class-determining structural feature determination were initially carried out for each tannin listed in Table 1 and have been reported elsewhere.^43,44^

### Condensed Tannins

**Figure 1:**
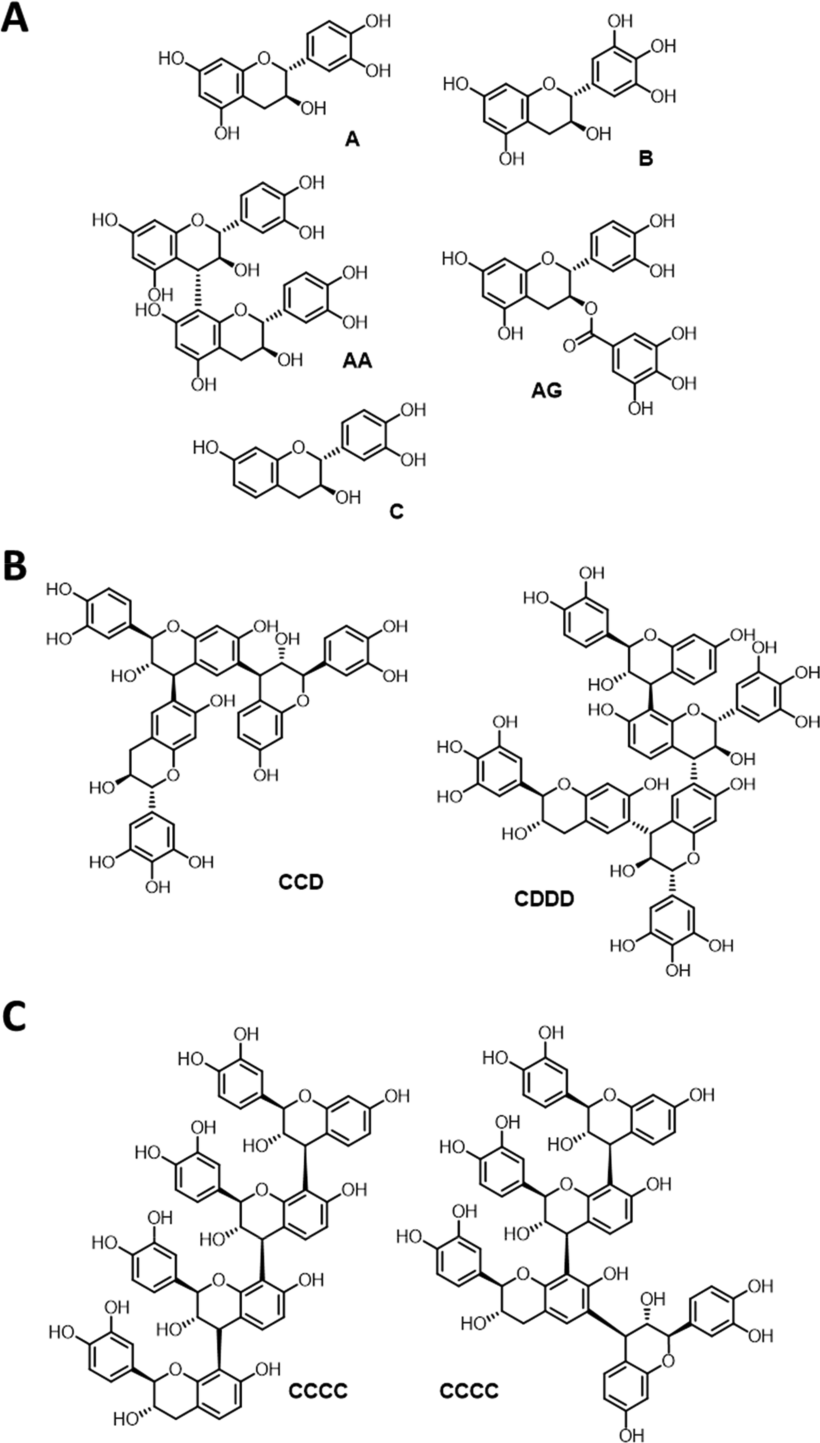
Structural representations of condensed/complex tannins used in this study: (A) OmniVin WG (***Vv***) and OmniVin 20R (***Vv*****-20**);^44^ (B) MIMOSA ATO ME (***Am***);^44^ and (C) *Schinopsis balansae* wood extract (***Sb*****W**).^43^ Letter code: A—(epi)catechin (in procyanidins), B—(epi)gallocatechin (in prodelphinidins), C—fisitinidol (in profisetidins), D—robinetinidol (in prorobinetinidins), and G—galloyl.

Structures of condensed tannins ***Vv***, ***Vv*****-20**, and ***Am*** are summarized in Figure 1. These three tannins were identified as mixtures of (epi)catechins and fisetinidols with some gallo(epi)catechin motifs in the case of ***Vv*** and ***Vv*****-20**; traces of *O*-gallates were found in both samples.^44^***Am*** is mainly composed of profisetidins (65%), with the remaining structures being prorobinetinidins (35%).^44^ Condensed tannin ***Sb*****W** has been characterized and structurally described before as well, and was found to represent a profisetidins.^43^

**Figure 2:**
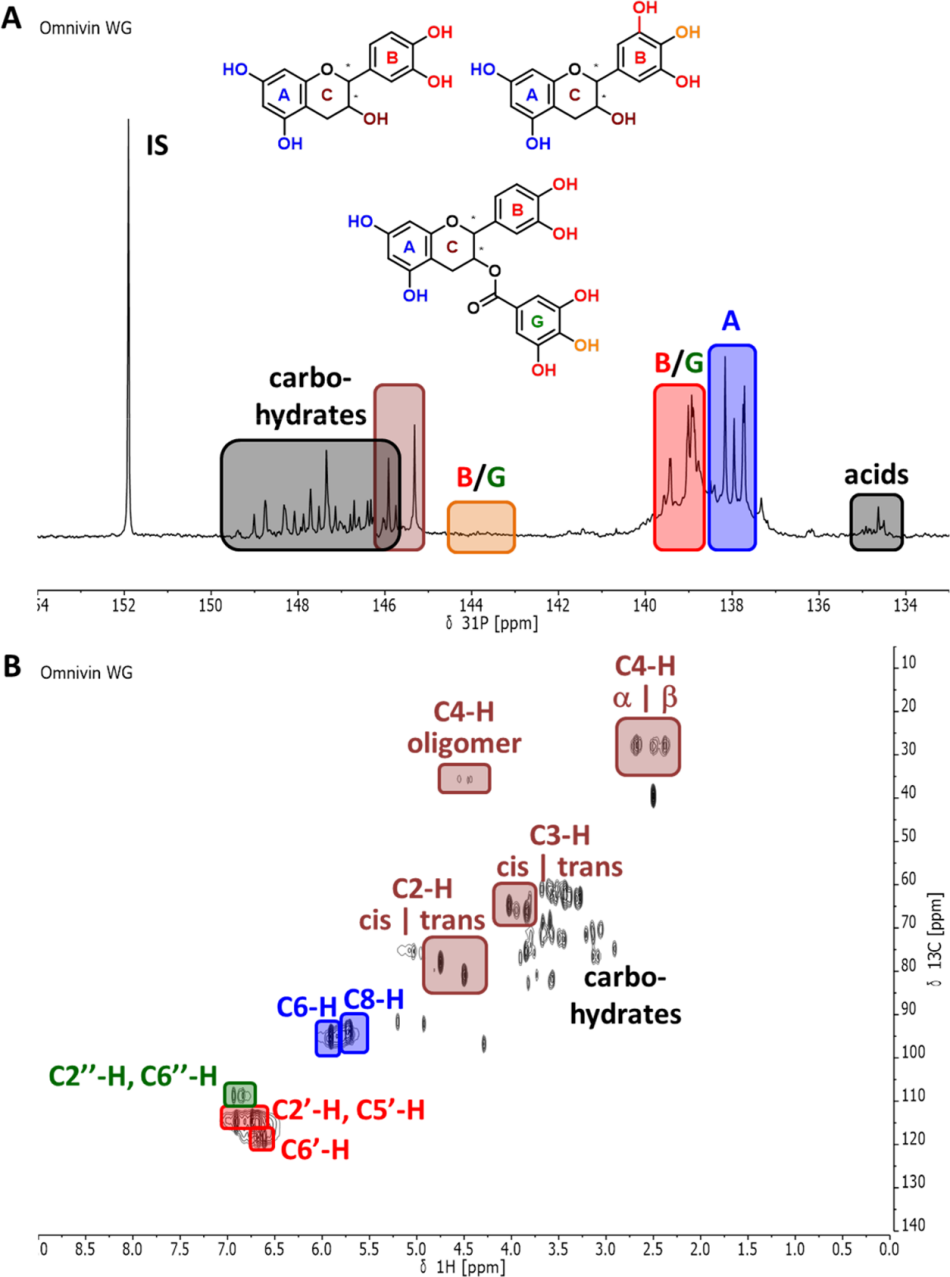
Structural analysis of ***Vv***: (A) Spectrum generated during quantitative ^31^P NMR analysis with the assignment of signals relative to rings A, B, and C, to catechin and epicatechin groups. as well as to gallates and (B) the HSQC spectrum with assignments of crucial cross-peaks.

Given the importance of structural features in the context of this work, and also in general for understanding and/or rationalizing the observed activity profiles on the basis of structural motifs, structure elucidation on the basis of the spectra obtained using quantitative ^31^P NMR (Figure 2A) and (qualitative) HSQC spectroscopic analyses (Figure 2B) shall be outlined here, in brief, once more for the case of ***Vv***.^44^

Analysis of the ^31^P NMR spectrum indicates, via the characteristics shifts indicated in Figure 2A,^43,45^ the presence of (epi)catechins, (epi)gallocatechins, and their gallate derivatives. This finding is confirmed by the HSQC spectrum, which additionally reveals the presence of low amounts of oligomeric species, via the cross-peak typical for “C4-H oligomers” indicated in the figure. Both ^31^P NMR and HSQC analyses indicate the presence of carbohydrate residues in the tannin.

Although HSQC analysis allows for identification of monomers, including stereochemical aspects, and eventual binding motifs within oligomeric structures, the results of the quantitative ^31^P NMR analyses are especially of importance in this work, since stoichiometries for the reactions and technical loadings as characteristic of the realized products are based on them. Data show that ***Vv***, ***Vv*****-20**, and ***Am*** contain comparable amounts of phenolic hydroxyl groups, and thus the anchoring points for functionalizations per gram of the material; ***Sb*****W** contains only half as many phenolic OH groups. The distribution of the phenolic OH groups across the various distinguishable types varies according to the main structural motif(s) present. The results obtained for various condensed tannins are listed in Table 2.

**Table 2:** Results of Quantitative ^31^P NMR Analyses of Phosphitylated Commercially Available Condensed Tannins Shown in Figure 1(43,44)

condensed tannins	aliphatic OH	pyrogallol OH^b^	gallate OH^b^	catechol OH^b^	A-ring OH	total phenol OH^c^	acidic OH
***Vv***	4.22	0.11	0.50	2.64	1.38	9.18	0.55
***Vv*-20**	2.57	0.00	0.25	3.06	3.51	10.9	0.47
***Am***	5.97	1.54	0.27	1.85	0.61	8.28	0.12
***Sb*W**	3.36	0.00	0.00	3.96	1.78	4.48	0.28

a Results are given in mmol/g; assignments are based on the literature reports.^43,44^

b Abundance of motifs as a whole, *i.e*., pyrogallol with 3 OH groups, catechol with 2 OH groups.

c Value over complete phenolic shift range (144.00–137.00 ppm).

Comparing the nuber of aliphatic hydroxyl groups to what could be expected on the basis of the identified structure allows for estimating sample purity, and indicates also in cases of ***Am*** and ***Sb*****W** the presence of carbohydrate impurities.^43,44^

### Hydrolyzable Tannins

**Figure 3:**
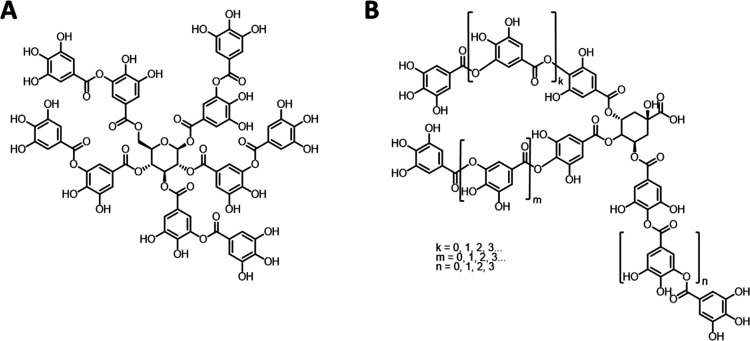
Structural representations of hydrolyzable tannins used in this study: (A) Tanal 01 (**Ta-01**); (B) Tanal 04 (**Ta-04**).^44^

The structure of hydrolyzable tannins **Ta-01** and **Ta-04** are shown in Figure 3. **Ta-01** represents a “typical” tannic acid, while **Ta-04** could be identified as a galloquinic acid derivative. A more detailed mass analysis of **Ta-04** by MALDI-ToF suggested a quinic acid core esterified with a total of 3–12 galloyl units.^44^ Most importantly with respect to the current work, hydroxyl group contents have been qualitatively and quantitatively assessed on the basis of quantitative ^31^P NMR spectroscopy.^44^ The results obtained for the two hydrolyzable tannins in this study are given in Table 3. Data indicate that the difference between the two samples in terms of the overall usable phenolic OH-group content is not very large, with **TA-01** providing approx. 12% more anchoring points for functionalization.

**Table 3:** Results of Quantitative ^31^P NMR Analyses of the Phosphitylated Commercially Available Hydrolyzable Tannins Shown in Figure 2^a^(44)

hydrolyzable tannins	aliphatic OH	internal gallate	terminal gallate	catechol OH	ortho-subst. phenol OH	total phenol OH^b^	acidic OH
**Ta-01**	0.59	2.20	2.51	3.27	4.58	13.5	0.22
**Ta-04**	0.92	2.21	1.84	3.38	3.06	11.9	0.15

a Results are given in mmol/g; assignments are based on the literature reports.^44^

b Value over complete phenolic shift range (144.00−137.00 ppm).

### Motifs for Functionalization

**Figure 4:**
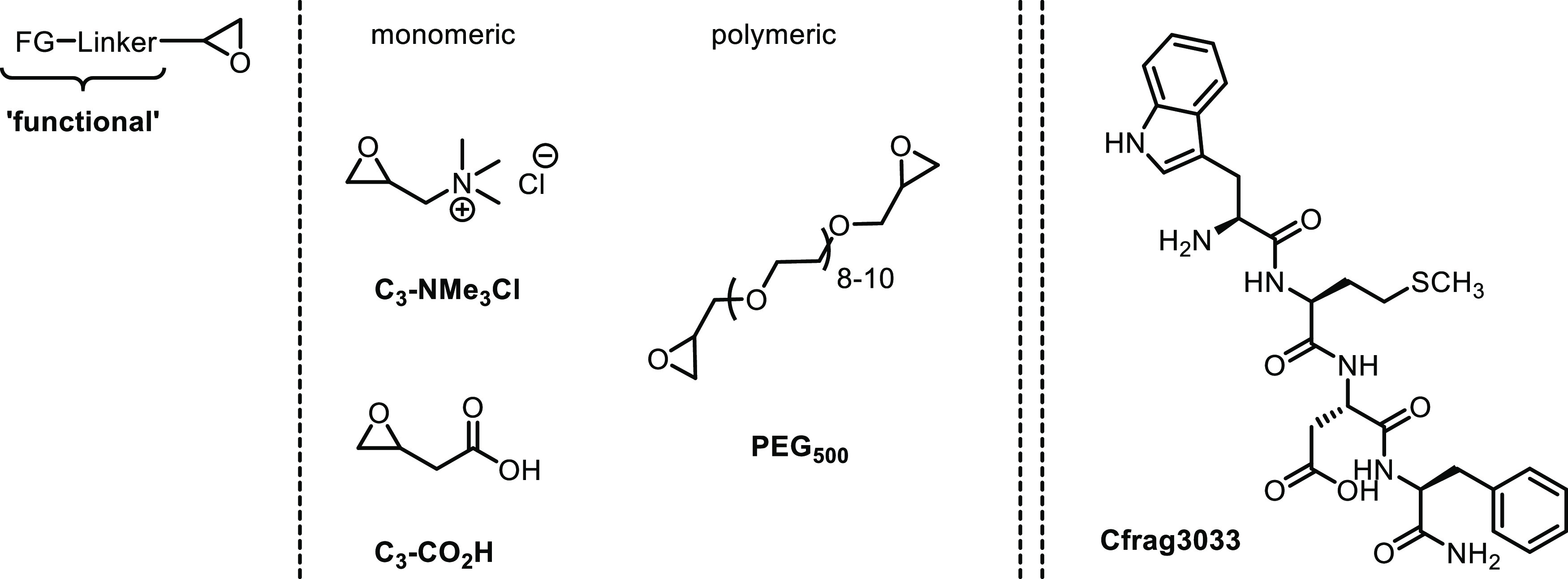
Epoxy-terminated monomeric “functionalities” **C**_**3**_**-NMe**_**3**_**Cl** and **C**_**3**_**-CO**_**2**_**H**, oligomeric bifunctional **PEG**_**500**_ and oligopeptide cholecystokinin fragment 30–33 (**Cfrag3033**) used for tannin functionalization.

Functional motifs to be added to the tannin backbones were chosen to confer to the tannin base structure motifs that would either enhance surface adhesion characteristics and alter their solubility profiles or enhance/confer bactericidal and/or bacteriostatic powers. Groups to be attached to the tannin backbones via relatively chemically stable phenol ethers^52^ include carboxylic acid groups, ammonium salts, and poly(ethylene glycol) motifs (Figure 4).

As a proof of concept, functionalized tannins were also converted subsequently to novel types of peptidomimetics, i.e., tannins carrying small peptide residues (Figure 4). Facile chemical routes were designed to allow for targeted tuning of macroscopic characteristics of the novel tannin-based substances via control of the degree of functionalization.

#### Functionalization of Tannins with Monomeric Functionalities

**Scheme 1:**
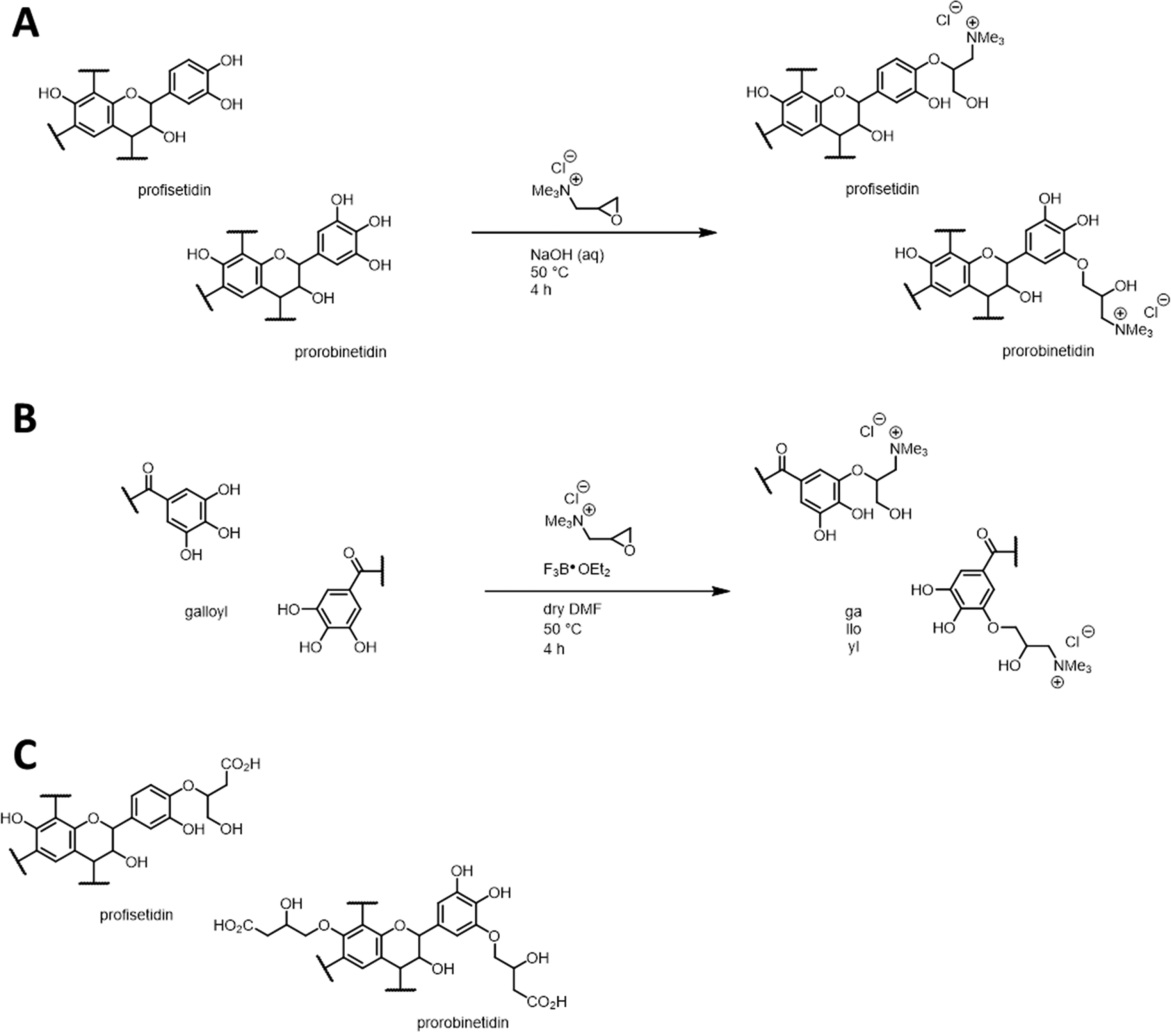
Exemplary syntheses of (A) ***Am*****C**_**3**_**NMe**_**3**_**Cl-0.1** using the SA-A method; (B) **Ta-01 C**_**3**_**NMe**_**3**_**Cl-0.1** via the SA-D method; and (C) structural motifs generated in ***Am*****C**_**3**_**CO**_**2**_**H-0.5** Exemplary structural motifs have been used for representation.

In an effort to develop reaction protocols with the lowest amount of organic solvents, following previous findings in the context of functionalization of lignins,^53^ the protocol for functionalizing condensed tannins has been based on the use of aqueous sodium hydroxide solutions to activate the phenolic OH groups for forming ether bonds by the ring-opening of an epoxide moiety present in the chosen functionalities, termed SA-A. Scheme 1A shows a typical reaction.

Sodium hydroxide was applied essentially in stoichiometric amounts for activating a defined, limited number of phenolic OH groups in various tannins; nevertheless, this approach was deemed unsuitable for hydrolyzable tannins. Background reactions like hydrolysis and transesterifications should be avoided. An alternative protocol using Lewis-acidic boron trifluoride diethyl etherate (F_3_B·OEt_2_) was thus established for activating the functional group-carrying epoxides in dry dimethylformamide (DMF); in the following, this protocol is referred to as SA-D. An exemplary reaction is shown in Scheme 1B.

Since the approach relied on the insights gained during the functionalization of lignins, optimization of conditions focused, after initial results, on the effects stemming eventually from significantly high concentrations and/or from the chosen reaction scale. The conditions described in the Experimental Section and thus used for generating Table 4 represent optimum conditions in terms of overall reproducibility. For obtaining a specific loading, a series of experiments leading to a sort of calibration that intrinsically accounts for the differences in reactivity and reaction conditions would be needed. This has not been done in this study, since the focus was on generating derivatized tannins for a very initial activity screening, such as to delineate whether an introduced functional group changes, especially, the biological properties.

**Table 4:** Results Obtained for the Functionalizations of Various Tannins with Monomeric Functionalities

entry	tannin	functional group (equiv)	synthetic approach^a^	work-up^b^	product	yield [%]	loading^c^ [%]
1	***Vv***		SA-A	WU-R	***Vv* blank-A**	72	
2		**C_3_-NMe_3_Cl** (0.1)	SA-A	WU-R	***Vv* C_3_NMe_3_Cl-0.1**	58	10
3		**C_3_-NMe_3_Cl** (0.5)	SA-A	WU-D	***Vv* C_3_NMe_3_Cl-0.5**	33	12
4		**C_3_-CO_2_H** (0.1)	SA-A	WU-R	***Vv* C_3_CO_2_H-0.1**	49	36
5		**C_3_-CO_2_H** (0.5)	SA-A	WU-D	***Vv* C_3_CO_2_H-0.5**	47	13
6	***Vv*-20**		SA-A	WU-R	***Vv*-20 blank-A**	47	
7		**C_3_-NMe_3_Cl** (0.1)	SA-A	WU-R	***Vv*-20 C_3_NMe_3_Cl-0.1**	46	14
8		**C_3_-NMe_3_Cl** (0.5)	SA-A	WU-D	***Vv*-20 C_3_NMe_3_Cl-0.5**	36	40
9		**C_3_-CO_2_H** (0.1)	SA-A	WU-R	***Vv*-20 C_3_CO_2_H-0.1**	45	6
10		**C_3_-CO_2_H** (0.5)	SA-A	WU-D	***Vv*-20 C_3_CO_2_H-0.5**	60	92
11	***Am***		SA-A	WU-R	***Am* blank-A**	90	
12		**C_3_-NMe_3_Cl** (0.1)	SA-A	WU-R	***Am* C_3_NMe_3_Cl-0.1**	44	n.n.^d^
13		**C_3_-NMe_3_Cl** (0.5)	SA-A	WU-D	***Am* C_3_NMe_3_Cl-0.5**	53	n.n^d^
14		**C_3_-CO_2_H** (0.1)	SA-A	WU-R	***Am* C_3_CO_2_H-0.1**	55	n.n.^d^
15		**C_3_-CO_2_H** (0.5)	SA-A	WU-D	***Am* C_3_CO_2_H-0.5**	27	n.n.^d^
16	***Sb*W**		SA-A	WU-P	***Sb*W blank-A**	84	
17		**C_3_-NMe_3_Cl** (0.5)	SA-A	WU-P	***Sb*W C_3_NMe_3_Cl-0.5**	55	n.n.^d^
18		**C_3_-CO_2_H** (0.5)	SA-A	WU-P	***Sb*W C_3_CO_2_H-0.5**	65	19^e^
19		**C_3_-CO_2_H** (1.2)	SA-A	WU-P	***Sb*W C_3_CO_2_H-0.5**	77	43^e^
20	**Ta-01**		SA-D	WU-R	**Ta-01 blank-D**	70	
21		**C_3_-NMe_3_Cl** (0.1)	SA-D	WU-R	**Ta-01 C_3_NMe_3_Cl-0.1**	40	n.n.^d^
22		**C_3_-NMe_3_Cl** (0.5)	SA-D	WU-D	**Ta-01 C_3_NMe_3_Cl-0.5**	87	23
23		**C_3_-CO_2_H** (0.1)	SA-D	WU-R	**Ta-01 C_3_CO_2_H-0.1**	24	<1
24		**C_3_-CO_2_H** (0.5)	SA-D	WU-D	**Ta-01 C_3_CO_2_H-0.5**	28	<1
25	**Ta-04**		SA-D	WU-R	**Ta-04 blank-D**	92	
26		**C_3_-NMe_3_Cl** (0.1)	SA-D	WU-R	**Ta-04 C_3_NMe_3_Cl-0.1**	26	n.n.^d^
27		**C_3_-NMe_3_Cl** (0.5)	SA-D	WU-D	**Ta-04 C_3_NMe_3_Cl-0.5**	15	15
28		**C_3_-CO_2_H** (0.1)	SA-D	WU-R	**Ta-04 C_3_CO_2_H-0.1**	69	n.n.^d^
29		**C_3_-CO_2_H** (0.5)	SA-D	WU-D	**Ta-04 C_3_CO_2_H-0.5**	20	n.n.^d^

a SA-A: synthesis using aqueous sodium hydroxide; SA-D: synthesis using F_3_B·OEt_2_ in dry DMF.

b WU-R: work-up using microporous resin (Amberlyst XAD); WU-D: work-up using dialysis bags; WU-P: work-up using precipitation and centrifugation.

c Determined via ^1^H NMR spectroscopy if not indicated otherwise; %-values represent the amount of functional groups per monomer unit of the tannin.

d Sample not sufficiently soluble under analysis conditions.

e Determined via quantitative ^31^P NMR spectroscopy after phosphitylation, %-values represent the total amount of consumed phenolic OH groups.

Scheme 1D and C show other structures realized using either the sodium hydroxide protocol or the boron trifluoride diethyl etherate protocol, respectively. Quantitative aspects of the realized tannin derivatives are summarized in Table 4.

Data indicate that functionalization, as such proceeded by and large reliably with both the two protocols established. Yields of isolated materials were moderate though across the various species realized, independent of the work-up procedure that was chosen and applied on the basis of the changes in the physicochemical properties that were to be expected on the basis of the type of functional group introduced. The results obtained do not obviously correlate with the type of functionality introduced or with the technical loading factors delineated for the various samples where possible (vide infra). Nevertheless, successful product formation was immediately evident in all cases by significant changes in the physicochemical characteristics of the novel substances with respect to starting tannins. This fact might in part explain material losses; depending on the tannin starting material and functional group added, different work-ups became necessary to account especially for the altered solubility profiles. A screening of methods principally suitable for isolating oligomeric and polymeric phenolics carrying eventually charged moieties resulted in two preferred methods for the isolation of derivatized tannins: (i) an adsorption–desorption protocol using Amberlyst as the microporous resin, termed WU-R, and (ii) a dialysis protocol using conventional dialysis bags with a low molecular weight cut-off of 1–1.5 kDa, termed WU-D.

**Figure 5:**
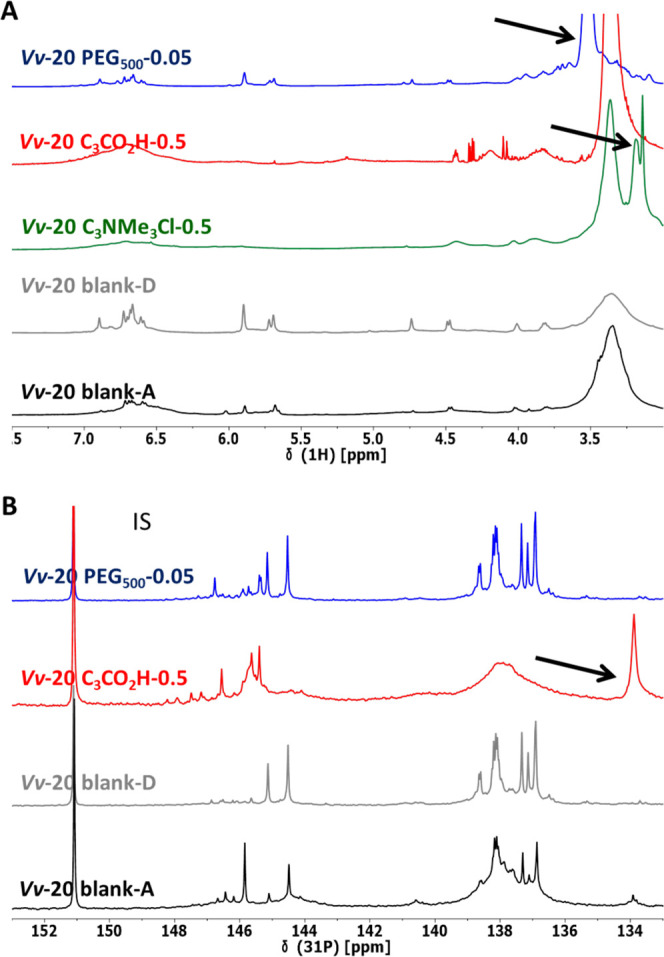
Comparison of (A) ^1^H NMR spectra and (B) ^31^P NMR spectra for various derivatives of ***Vv*****-20** (N.B: ***Vv*****-20 C**_**3**_**NMe**_**3**_**Cl-0.5** was not soluble under standard ^31^P NMR analysis conditions.). Arrows indicate the most characteristic peak of the introduced functionality. Legend: blank-A—reisolated tannin exposed to sodium hydroxide in water; blank-D—reisolated tannin exposed to Lewis-acid in DMF.

Generally, the successful transformation of various tannins into functional derivatives could be qualitatively confirmed using either ^1^H NMR or ^31^P NMR spectroscopy. Ammonium groups generated a new, characteristic signal at δ = 2.90 ± 0.05 ppm in the ^1^H NMR spectra (Figure 5A), while the addition of the carboxylic acid motif could be clearly monitored by an increase of the peak corresponding to the phosphitylated carboxylic OH in the ^31^P NMR spectra of phosphitylated samples (Figure 5B). Figure 4 shows a general comparison between the blanks obtained for tannin ***Vv*****-20** by different functionalization protocols and representative derivatives realized. Other signals attributable to the different functionalities introduced into the various tannins can eventually be identified and characterize the novel tannin derivatives as such. These peaks are, however, not very well observable, let alone reliably quantifiable in all tannin cases due to signal overlaps.

MALDI-TOF was used for additional verification of the formation of the desired products. Starting from the conditions established before for the MALDI-ToF analyses of the tannins used here,^44^ it was possible to acquire mass spectra of most of the derivatives; exceptions were met with PEG-ylated hydrolyzable tannins, Quebracho samples have not been analyzed. Figures S1–S10 in the Supporting Information show representative MALDI-ToF spectra of functionalized tannins. Table 5 shows a selection of identified species; Tables S1 and S2 in the Supporting Information list these and further identified derivatives for condensed and hydrolyzable tannins, respectively. Overall, detectable species indicate that the functionalization of various molecules was partial in terms of OH groups as intended, and thus corresponds to the results obtained on the basis of NMR analysis.

**Table 5:** MALDI-ToF Analysis of Functionalized Condensed and Hydolyzable Tannins^a^3

			assignment		
functionalized tannin	observed mass peak [Da]	calculated mass [Da]	base structure	functional	# functional
***Vv*-20 C_3_NMe_3_Cl-0.5**	408.7	407.5	B	C_3_NMe_3_^+^	1
	697.0	695.8	AA		1
	848.9	847.9	AAG		1
	985.4	984.1	AAA		1
***Vv*-20 C_3_CO_2_H-0.5**	498.3	497.5	A + Na^+^	C_3_CO_2_H	2
	651.0	649.6	AG + Na^+^		1
***Vv*-20 PEG_500_-0.25**	812.4	809.3	A + Na^+^	PEG_500_ n.c.^b^	1
	816.3	813.3	A + Na^+^	PEG_500_ c.^b^	1
	828.0	829.3	B + Na^+^	PEG_500_ c.^b^	1
	943.8	943.4	AG + H^+^	PEG_500_ n.c.^b^	1
	977.2	977.4	BG + H^+^	PEG_500_ n.c.^b^	1
***Am* C_3_NMe_3_Cl-0.5**	697.9	695.8	DD	C_3_NMe_3_	1
	811.3	813.0	DD		2
	1099.3	1101.3	DDD		2
***Am* C_3_CO_2_H-0.5**.	496.8	497.5	D + Na^+^	C_3_CO_2_H	2
	512.7	513.5	B + Na^+^		2
***Am* PEG_500_-0.25**	1061.9	1063.6	CD + H^+^	PEG_500_ c.^b^	1
	1093.1	1097.6	DD + H^+^	PEG_500_ c.^b^	1
	1238.3	1238.7	CDG + Na^+^	PEG_500_ c.^b^	1
		1239.7	CCG + Na^+^	PEG_500_ n.c.^b^	1
**Ta-01 C_3_NMe_3_Cl-0.5**	441.5		L8 + 1Na^+^	C_3_NMe_3_	3
**Ta-01 C_3_CO_2_H-0.5**	999.2	995.8	L4 + H^+^	C_3_CO_2_H	2
	1609.3	1604.2	L8 + H^+^		2
**Ta-04 C_3_NMe_3_Cl-0.5**	394.1	391.4	Q4 + Na^+^	C_3_NMe_3_	3
	458.6	461.5	Q1 + Na^+^		1
	679.4	681.1	Q6 + 2Na^+^		1
**Ta-04 C_3_CO_2_H-0.5**	446.0	448.4	Q1 + H^+^	C_3_CO_2_H	1
	622.7	624.8	Q9 + 3H^+^		1
	927.2	926.7	Q4 + Na^+^		1
	1079.9	1078.8	Q5 + Na^+^		1
	1232.6	1230.9	Q6 + Na^+^		1
	1385.1	1383.0	Q7 + Na+		1

a For letter codes of identified monomeric tannin building blocks refer to Figures 2 and S11, and for functionalities refer to Figure 3.

b c.—crosslinking; n.c.—non crosslinking.

Unlike qualitative analysis, determination of technical loading factors as means of quantifying the structural modification turned out to be difficult due to solubility issues under conditions that would otherwise allow for both quantitative ^1^H spectroscopy and ^31^P NMR spectroscopy, inhibiting in some cases even a rough quantitative analysis. Quantification could be achieved in case of adequate sample solubility by performing ^1^H NMR analysis in deuterated dimethyl sulphoxide using phthalimide as the internal standard; the typical shift of the C–H in phthalimide at δ = 7.86 ppm proved to be rather isolated and thus can be easily integrated accurately while being still positioned at sufficient vicinity to characteristic protons of the analytes. Measurements against the internal standard were combined with a normalization approach on the basis of the fact that protons securely not affected by the functionalization, i.e., the aromatic protons: the integral value for the aromatic region (7.50–5.50 ppm) of the functionalized tannin was normalized with respect to the corresponding integral of the blank sample to determine a normalization factor. The integral of the aliphatic proton region (5.50–2.88 ppm) of the functionalized samples also containing the protons of the introduced functional group was corrected for the normalization factor and then divided by the total number of aliphatic protons present in the functionalized tannin, i.e., the sum of aliphatic protons from the base tannin and the introduced functional group. Technical loadings determined via ^1^H NMR spectroscopy were reported as the relative number of functional groups per monomeric unit compared to the blank, as listed in Table 4.

In some cases, in which the determination of loadings was not possible using ^1^H NMR, technical loadings could be approximated via quantitative ^31^P NMR spectroscopy after phosphitylation of samples. Estimated technical loadings as reported in Table 2 represent the total consumed phenolic OH groups compared to the blank. An exact determination of loadings is not possible with this approach, since the addition of the chosen functional groups brings with it a change in the molecular weight. For example, in the case of a trimeric ***Am***, the addition of an ammonium functionality represents a 20% increase in the molecular weight of the structure. In light of the way quantitative data are derived via the ^31^P NMR method, a more significant error compared to the one routinely encountered for quantitative ^31^P NMR analysis, i.e., around 0.02 mmol/g,^47^ is encountered in these cases; nevertheless, technical loadings determined by this approach are more than suitable for reliably indicating trends.

Comprehensive analysis of the results do not indicate a fully homogeneous picture. In the case of condensed tannins, yields of isolated functionalized materials are moderate, independent of the momomeric functionality attached. Loading factors correlate only roughly with the added equivalents of functional groups; this aspect, seen independently of the method used for deriving technical loading indications, could not be fully resolved yet. The amounts of isolated materials or correlation of loading factors with the amount of functional groups used do not seem to depend on the tannin size (compare Figure 1). This interesting finding suggests eventually an expectably more complex interplay between electronic and steric effects that will differ across tannin species, of course, but also more subtle between different regioisomers of the same oligomeric tannin species, e.g., between tetrameric example structures shown in Figure 1C.

The volitional simplicity of the experimental set-up does not allow for stabilizing a reliable “reactivity ranking” across the various phenolic OH groups present in different tannins, fewer regioisomers; this nevertheless interesting and important aspect is currently subject to ongoing investigations in our groups.

#### Functionalization of Tannins with an Oligomeric PEG-Crosslinker

**Figure 6:**
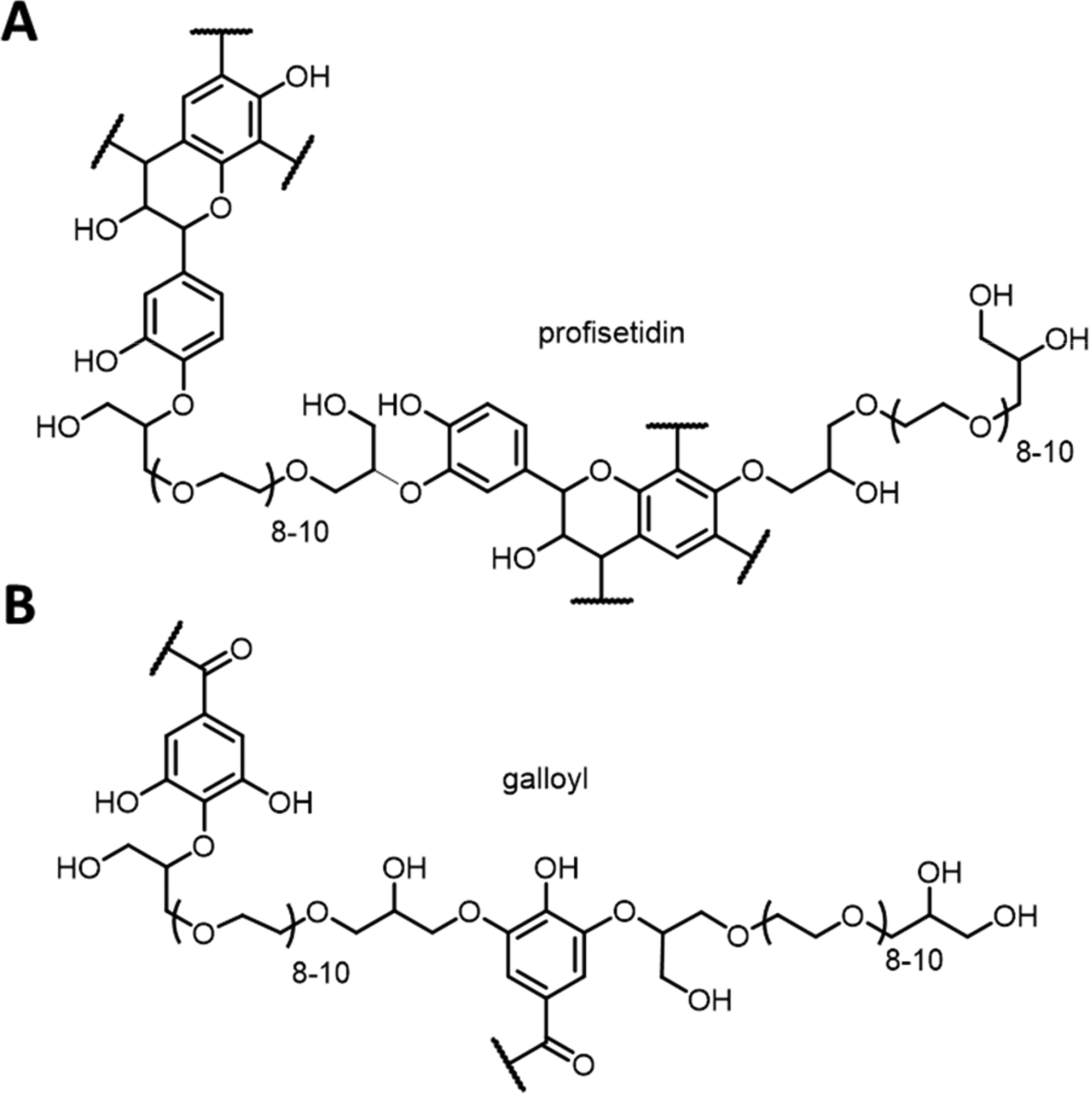
Representative structures generated by cross-linking of (A) ***Am*** as condensed and (B) **Ta-01** as hydrolyzable tannin with bifunctional **PEG**_**500**_. Exemplary structural motifs have been used for representation.

To modify the inherent hydrophilicity of the tannins under study and to generate an amphiphilic “tannin network,” the second route of tannin functionalization consisted of the attachment of a hydrophilic oligomeric poly(ethylene glycol) diglycidyl ether, **PEG**_**500**_ (Figure 2). The choice of this specific polymer is related to its biocompatibility and extensive use in home care (hard and soft surface detergents) and personal care (hair softeners) products. Condensed tannins ***Vv***, ***Vv*****-20**, and ***Am***, as well as hydrolyzable tannins **Ta-01** and **Ta-04**, were intermolecularly cross-linked under concomitant ether formation using the **PEG**_**500**_ functionality. Reactions were exclusively performed in dry DMF and catalyzed by boron trifluoro etherate in all cases for this functionalization, i.e., also in the case of condensed tannins ***Vv***, ***Vv-20***, and ***Am*** that are stable under the alkaline conditions used before. Interestingly, overall superior solubilities were achieved in DMF throughout the entire reaction sequence including the work-up. The results are summarized for all co-polymerized tannins in Table 6; an exemplary structure for condensed tannins is given in Figure 6A, and the common structural aspect of hydrolyzable tannins is shown in Figure 6B.

**Table 6:** Results Obtained for the Cross-Linking of Condensed Tannins ***Vv***, ***Vv*****-20**, and ***Am*** and Hydrolyzable Tannins **Ta-01** and **Ta-04** with Polymeric Functionalities **PEG**_**500**_ Using Lewis-Acidic F_3_B·OEt_2_ in Dry DMF

tannin	functional unit (equiv)	synthetic approach^a^	work-up^b^	product	mass return [%]	loading^a^ [%]
***Vv***	**PEG_500_** (0.05)	SA-D	WU-R	***Vv* PEG_500_-0.05**	50	5
	**PEG_500_** (0.25)		WU-D	***Vv* PEG_500_-0.25**	41	10
***Vv*-20**	**PEG_500_** (0.05)	SA-D	WU-R	***Vv*-20 PEG_500_-0.05**	45	6
	**PEG_500_** (0.25)		WU-D	***Vv*-20 PEG_500_-0.25**	26	35
***Am***	**PEG_500_** (0.05)	SA-D	WU-R	***Am* PEG_500_-0.05**	48	4
	**PEG_500_** (0.25)		WU-D	***Am* PEG_500_-0.25**	15	22
**Ta-01**	**PEG_500_** (0.05)	SA-D	WU-R	**Ta-01 PEG_500_-0.05**	30	14
	**PEG_500_** (0.25)		WU-D	**Ta-01 PEG_500_-0.25**	23	53
**Ta-04**	**PEG_500_** (0.05)	SA-D	WU-R	**Ta-04 PEG_500_-0.05**	44	n.n.^b^
	**PEG_500_**(0.25)		WU-D	**Ta-04 PEG_500_-0.25**	33	18

a Determined by ^1^H NMR spectroscopy based on functional monomer units.

b A reliable determination of the actual loading was not possible due to limited solubility of the sample.

Products were generally isolated in acceptable yields. Product formation was monitored by ^1^H NMR analysis and proton spectra were also used for the estimation of the technical loading as described before (Table 6). Most interestingly, generally clearer trends are found when the various tannins were cross-linked with oligomeric **PEG**_**500**_ for the generation of novel types of block-copolymers. Yields drop for all products occurred with higher equivalents of PEG, indicating significantly higher hydrophilicity as planned. The determined loading factors correlate in terms of trends with the added equivalents of bifunctional **PEG**_**500**_; these trends go across the different tannin classes.

#### Peptidic Derivatization of **SbW** with Peptides

**Scheme 2:**
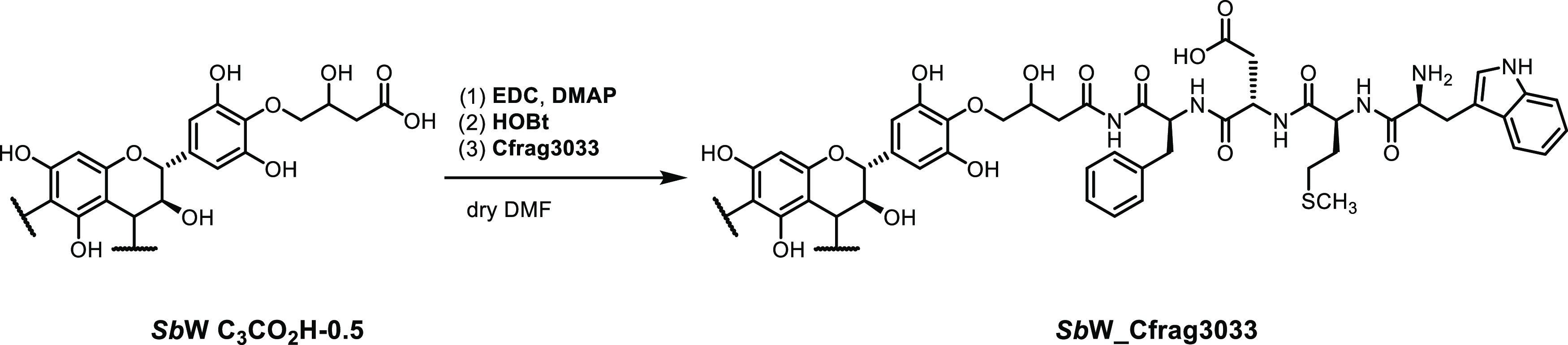
**:** Coupling Reaction Furnishing ***Sb*****W_Cfrag3033** Starting from ***Sb*****W C**_**3**_**CO**_**2**_**H-0.5**

As a proof of concept for envisaged applications of differently functionalized tannins as shell materials for tannin nano- and microcapsules for biomedical applications, a peptide sequence was attached to an oligomeric condensed tannin. Cholecystokinin fragment 30–33 (**Cfrag3033**) were chosen as commercially available oligopeptide with chemically interesting complexity. *Schinopsis balansae* wood extracts, ***Sb*****W**, structurally characterized in an earlier study,^43^ was first functionalized with **C**_**3**_**-CO**_**2**_**H** to display a C-terminus for traditional coupling reactions on a flexible linker. The activation of the attached carboxylic acid was achieved in dry DMF using *N*-ethyl-*N*′-(3-dimethylaminopropyl)carbodiimidehydrochloride (**EDC**); subsequent transesterification with *N*-hydroxybenzotriazole (**HOBt**) and addition of **Cfrag3033** then resulted in peptide decorated ***Sb*****W**. Scheme 2 shows the reaction sequence and the product ***Sb*****W_Cfrag3033**.

**Figure 7:**
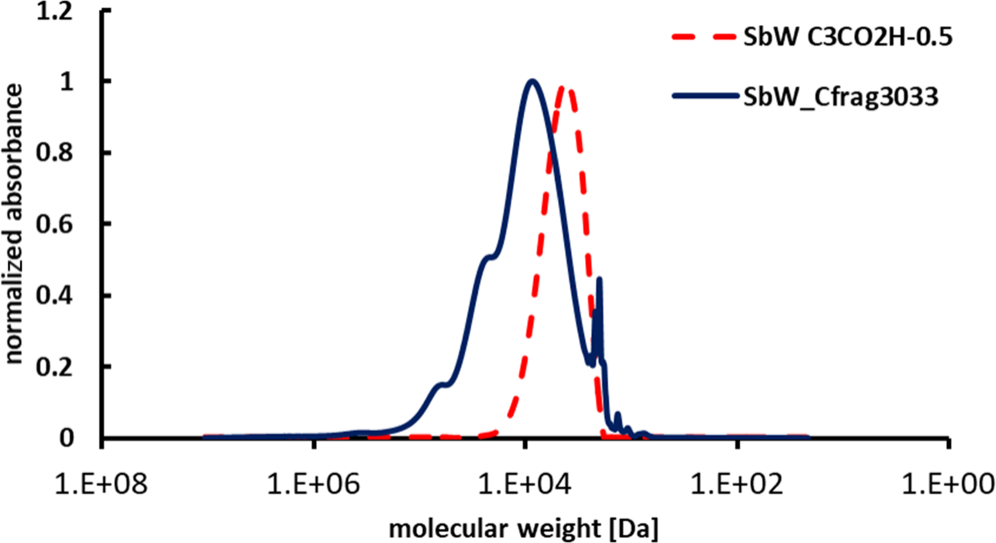
Overlay of GPC analysis of ***Sb*****W C**_**3**_**CO**_**2**_**H** and ***Sb*****W_Cfrag3033**.

GPC measurements turned out to be a reliable means for determining whether the linking was successful; in both FT-IR and ^1^H NMR analyses a mere mixture of the two compounds would not be distinguishable from a successfully formed product. GPC elution profiles indicated a successful addition of the peptide moiety to the polyphenolic tannin structure in the form of a clear shift toward higher molecular weights, i.e., from Mn = 3300 Da (polydispersity (PDI) = 2.6) to Mn = 4100 Da (PDI = 3.3), when monitoring at the typical absorbance maximum of λ = 280 nm (Figure 7) for polyphenols. These Mn-values, although probably slightly overestimating the molecular weights of the samples, indicate that in average one or two peptide units are added to a tannin core structure, eventually leaving some introduced carboxyl functionalities free. This first, not fully optimized successful proof-of-concept synthesis of a tannin-peptide fragment represents an important step toward the use of tannins in biomedical applications.

## Conclusions

Generally applicable methodologies for the functionalization of various condensed and hydrolyzable tannins with small functional groups introducing permanent or inducible charges have been devised. Condensed tannins could be derivatized in basic aqueous solutions, while Lewis-acid catalysis in anhydrous DMF was applied for hydrolyzable tannins. Different protocols were developed for the isolation of the differently functionalized tannins, and the best results were obtained using either an exchange resin or a dialysis protocol. Functionalizations could be realized at varying degrees of technical loadings, i.e., the amounts of introduced untypical tannin functionalities per number of phenolic hydroxyl groups. The same strategy was found suitable for the synthesis of polyethylene glycol-functionalized tannin copolymers. Condensed tannin functionalized with carboxylic acid moieties could be converted into a tannin–oligopeptide hybrid.

The realized tannins have been tested in specific antibiofilm experiments. The interesting results obtained will be published in due course.

## Supporting Information Available

Images of the MALDI-ToF chromatograms and Table listing the identified species (PDF)

## Abbreviations Used

***Am***: *Acacia mearnsii*
**C**_**3**_**-CO**_**2**_**H**: 2-oxiranylacetic acid
**C**_**3**_**-NMe**_**3**_**Cl**: glycidyltrimethylammonium chloride
**Cfrag3033**: cholecystokinin fragment 30–33
**Cl-TMPD**: 2-chloro-4,4,5,5-tetramethyl-1,3,2-dioxaphospholane
**DMAP**: *N*,*N*-dimethylaminopyridine
DMF: *N*,*N*-dimethyl formamide
DMSO: dimethyl sulfoxide
**EDC**: 1-ethyl-3-(3-dimethylaminopropyl) carbodiimide
**F**_**3**_**B·OEt**_**2**_: boron trifluoride diethyl etherate
***e*****-HNDI**: *N*-hydroxy-5-norbornene-2,3-dicarboxylic acid imide
**HOBt**: 1-hydroxybenzotriazol
**PEG**_**500**_: poly(ethylene glycol) diglycidyl ether (Mn = 500 Da)
**RT**: room temperature
SA-A: synthesis using aqueous sodium hydroxide
SA-D: synthesis using F_3_B·OEt_2_ in dry DMF
***Sb*****W**: *Schinopsis balansae* wood extract
**Ta**: Tanal tannin
***Vv***: *Vitis vinifera* tannin
WU-D: work-up using dialysis bags
WU-R: work-up using microporous resin (Amberlyst)
WU-P: work-up using precipitation and centrifugation
