# Supplementary material for: Chemical Derivatization of Commercially Available Condensed and Hydrolyzable Tannins
Source: ACS Sustain Chem Eng. 2021 Jul 16;9(30):10154–66. doi: 10.1021/acssuschemeng.1c02114 (PMC8411582; doi:10.1021/acssuschemeng.1c02114)
Supplement: Supplementary file 1 — sc1c02114_si_001.pdf [file sc1c02114_si_001.pdf]

Supporting Information

Chemical Derivatisation of Commercially Available Condensed and Hydrolysable Tannins

LiLi Zhen[a,b], Heiko Lange[b,c,‡,*] Luc Zongo[a], and Claudia Crestini[b,d,‡,*]

a University of Rome 'Tor Vergata', Department of Chemical Science and Technologies, Via della Ricerca Scientifica, 00133 Rome, Italy

b CSGI - Center for Colloid and Surface Science, Via della Lastruccia 3, 50019 Sesto Fiorentino, Italy

c University of Milano-Bicocca, Department of Earth and Environmental Sciences, Piazza della Scienza 1, 20126 Milano, Italy

d Ca' Foscari University of Venice, Department of Molecular Science and Nanosystems, Via Torino 155, 30170 Venice Mestre, Italy

‡ Affiliated with a *via* NAST – Nanoscience & Nanotechnology & Innovative Instrumentation Center.

* Corresponding authors: heiko.lange@unimib.it, claudia.crestini@unive.it

Number of pages: 9

Number of tables: 2

Number of figures: 11

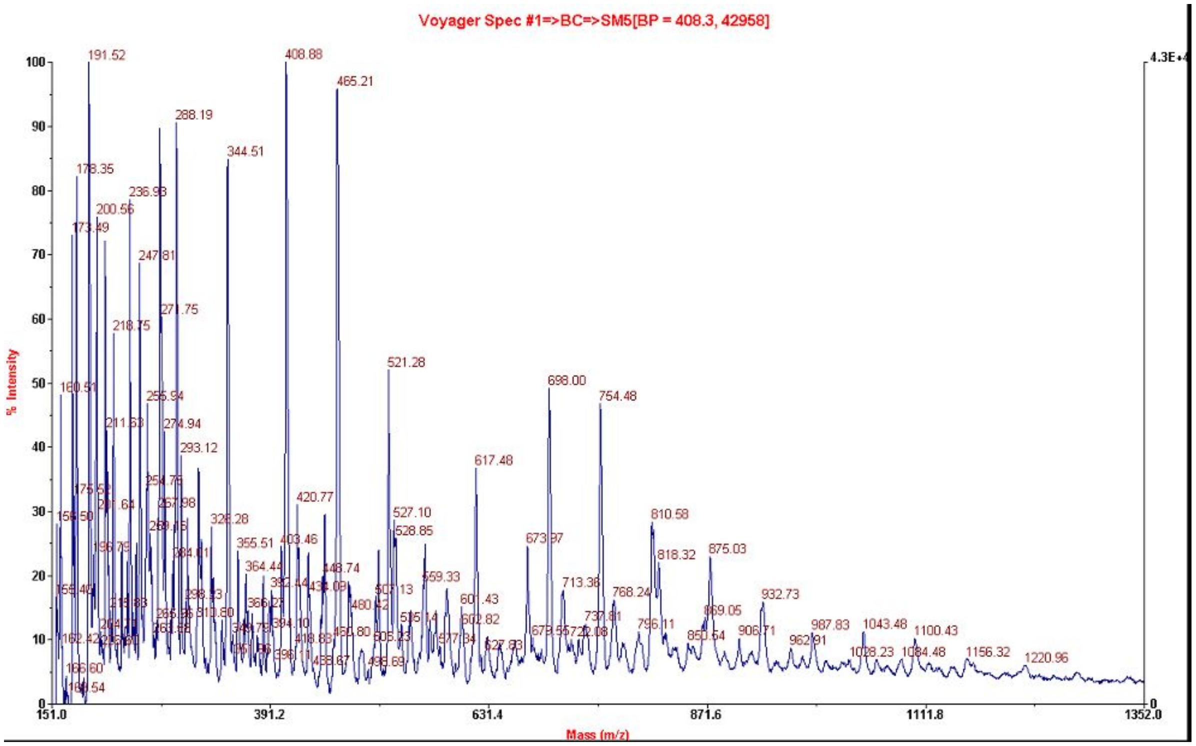

Figure S1: MALDI-ToF spectrum of ***Vv*-20 C_3NMe_3Cl-0.5**.

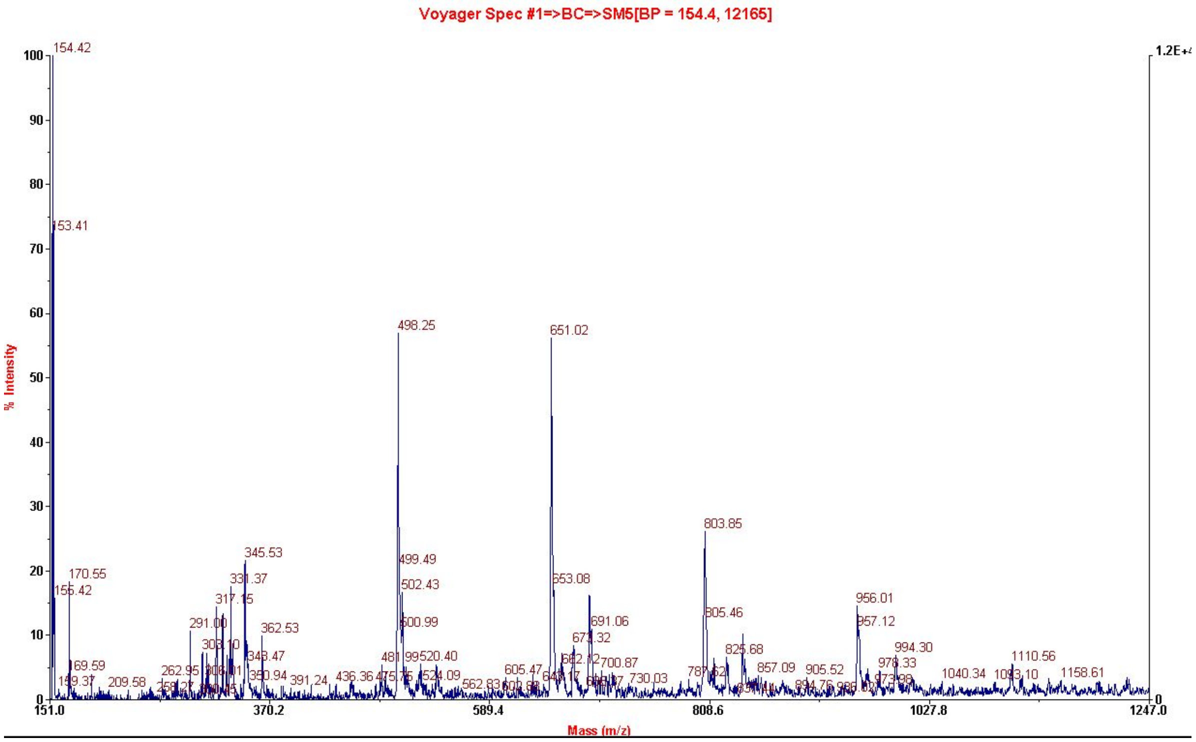

Figure S2: MALDI-ToF spectrum of ***Vv*-20 C_3CO_2H-0.5**.

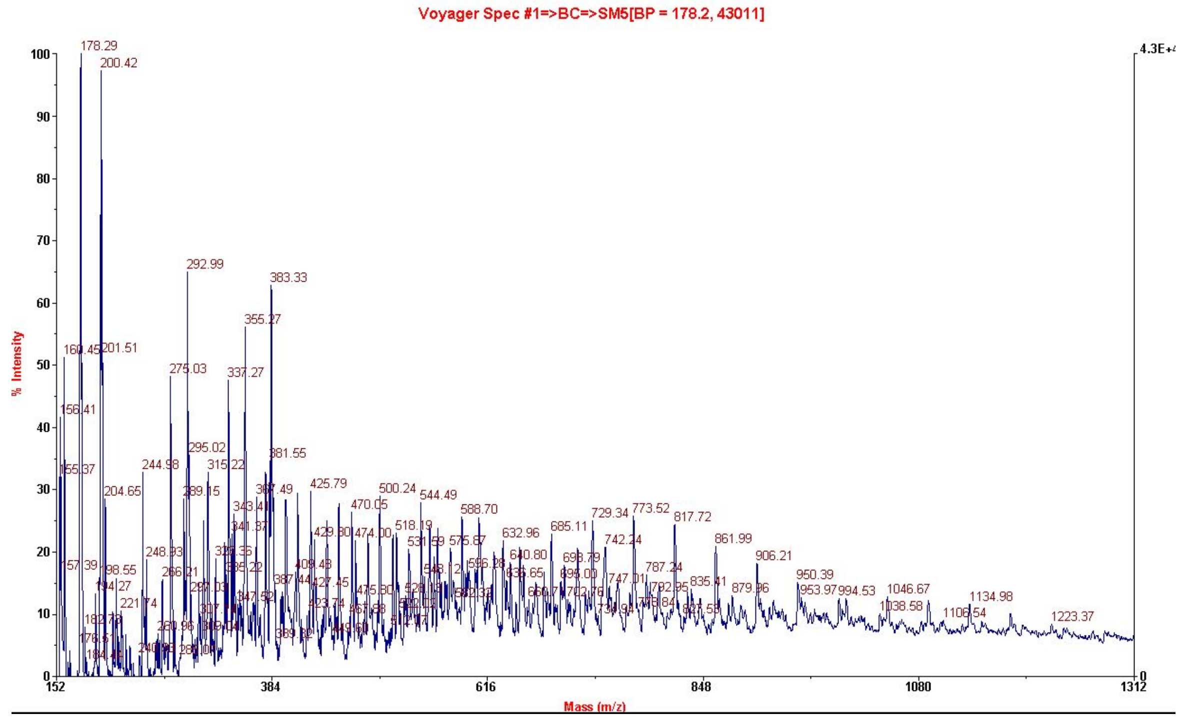

Figure S3: MALDI-ToF spectrum of ***Vv*-20 PEG$_{500}$-0.25**.

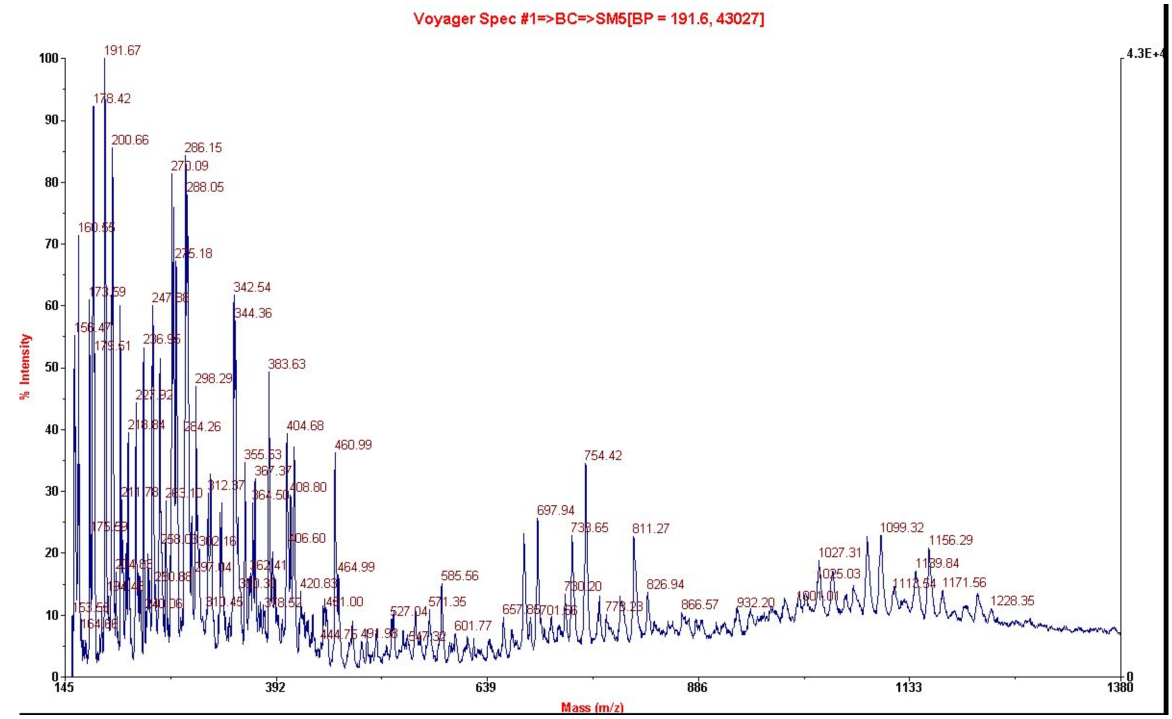

Figure S4: MALDI-ToF spectrum of ***Am* C$_3$NMe$_3$Cl-0.5**.

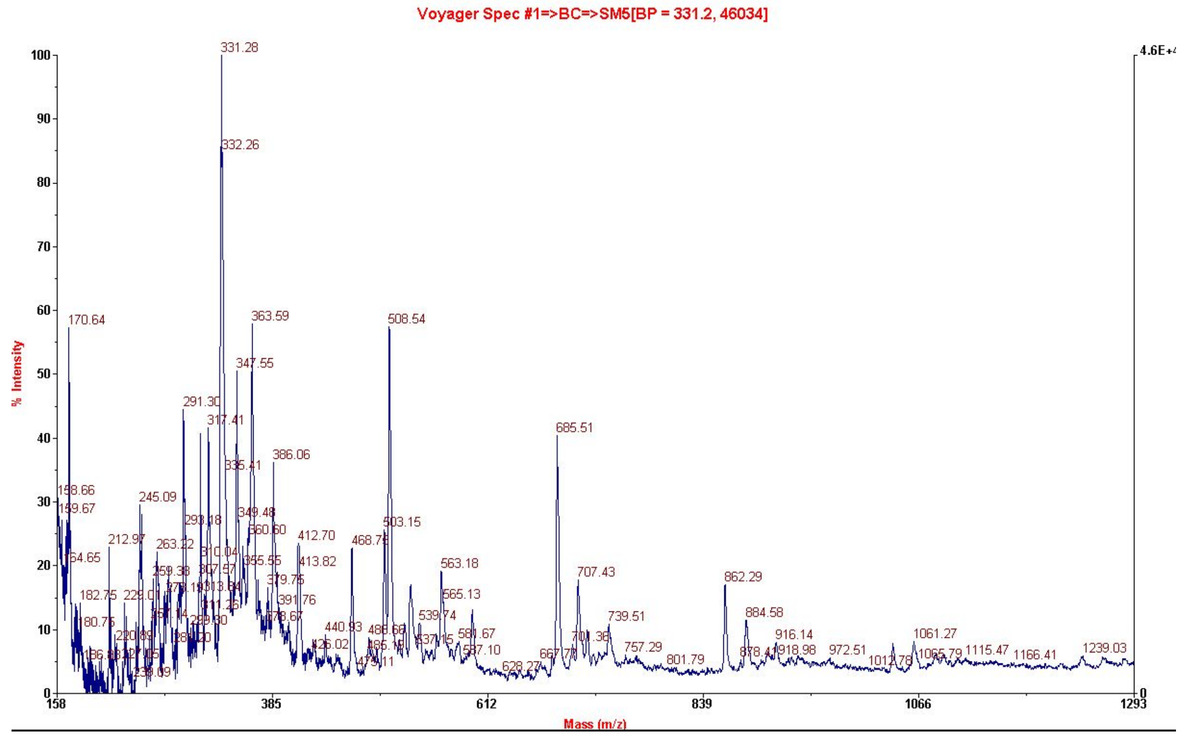

Figure S5: MALDI-ToF spectrum of ***Am*** **C_3CO_2H-0.5**.

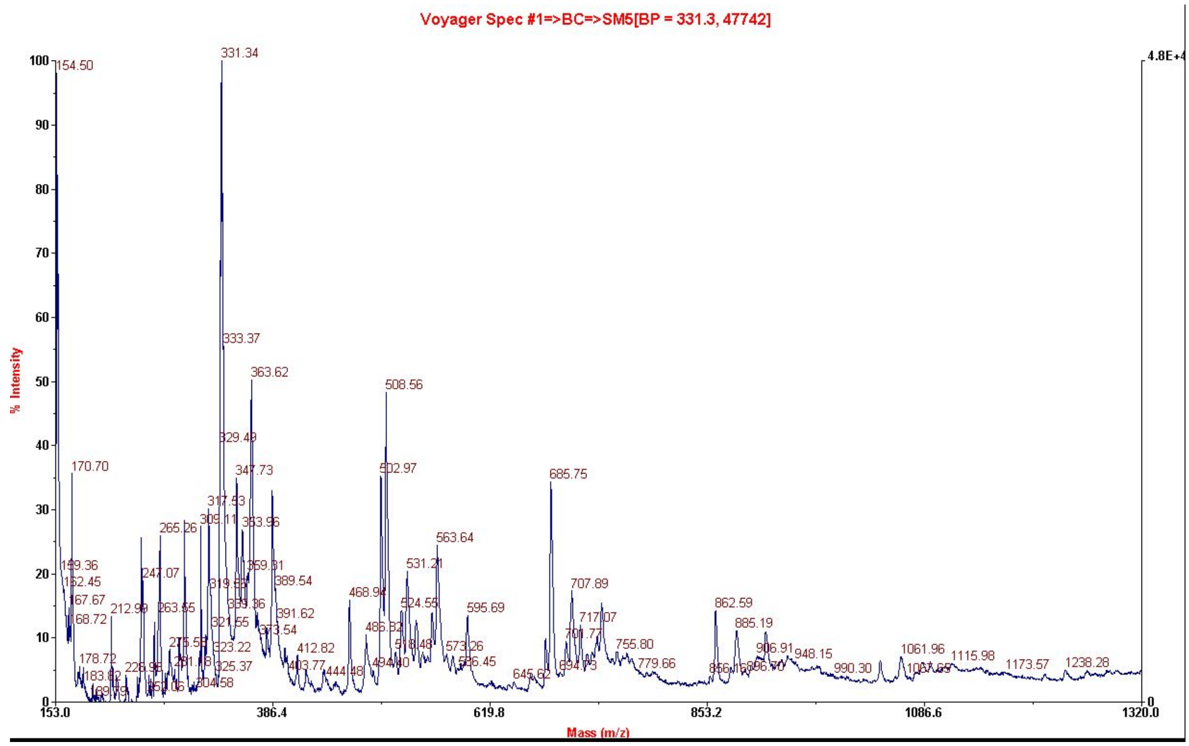

Figure S6: MALDI-ToF spectrum of ***Am*** **PEG_{500}-0.25**.

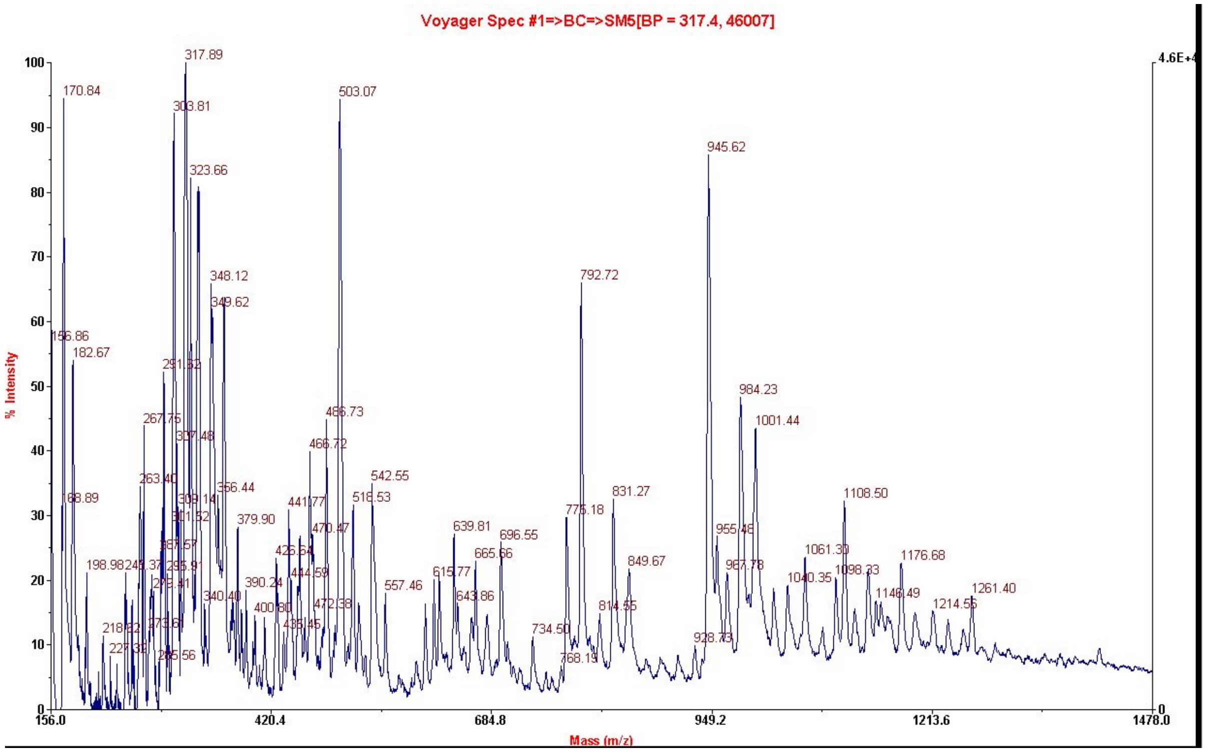

Figure S7: MALDI-ToF spectrum of **Ta-01 C_3NMe_3Cl-0.5**.

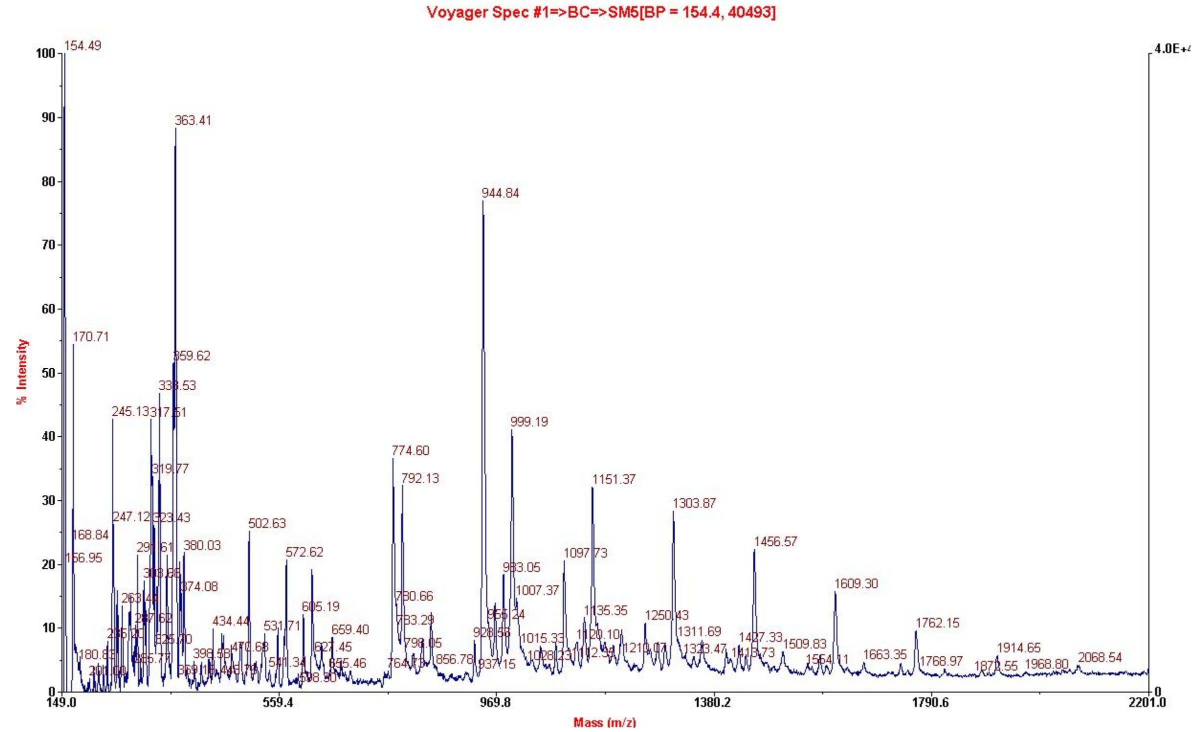

Figure S8: MALDI-ToF spectrum of **Ta-01 C_3CO_2H-0.5**.

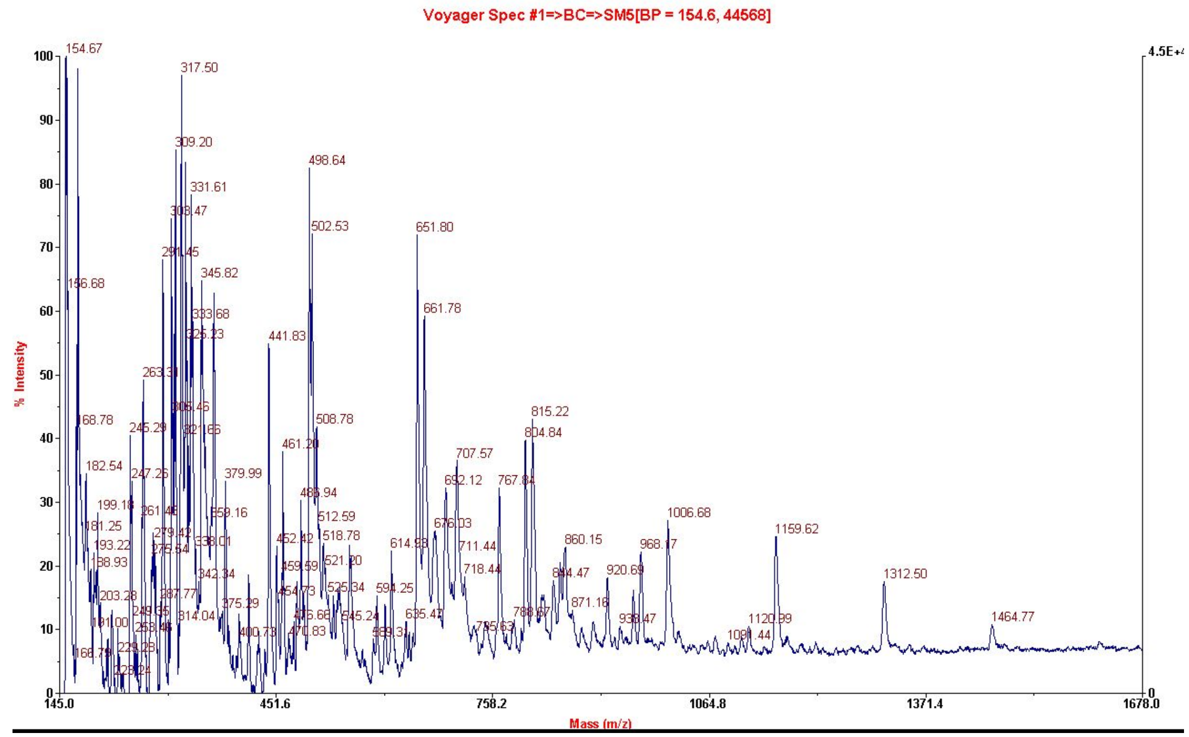

Figure S9: MALDI-ToF spectrum of **Ta-04 C_3NMe_3Cl-0.5**.

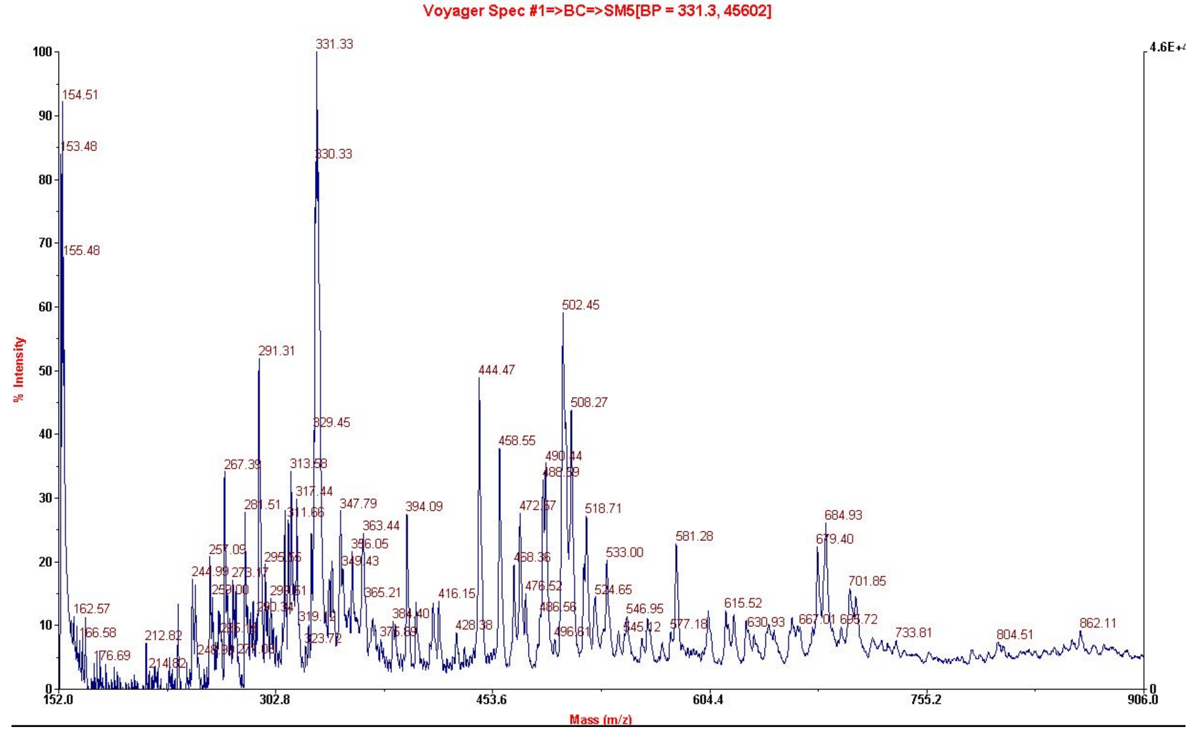

Figure S10: MALDI-ToF spectrum of **Ta-04 C_3CO_2H-0.5**.

Table S1: MALDI-ToF analysis of functionalised condensed tannins. For letter codes of identified monomeric tannin building blocks refer to Figure S11, for functionals to Figure 3.

**Functionalised tannin**	**Observed mass peak [Da]**	**Calculated mass [Da]**	**Assignment**		
			base strucure	funtional	# functional
***Vv*-20 C_3NMe_3Cl-0.5**	408.7	407.5	B	$C_3NMe_3^+$	
	697.0	695.8	AA		1
	848.9	847.9	AAG		1
	985.4	984.1	AAA		1
	1276.2	1272.4	AAAA		1
***Vv*-20 C_3CO_2H-0.5**	412.6	410.4	$B+Na^+$	C_3CO_2H	1
	498.3	497.5	$A+Na^+$		2
	651.0	649.6	$AG+Na^+$		1
	803.9	807.8	$AA+Na^+$		2
	907.2	910.9	$AA+Na^+$		3
	956.0	959.9	$AAG+Na^+$		2
***Vv*-20 PEG_{500}-0.25**	812.4	809.3	$A+Na^+$	PEG^{500} n.c.[a]	1
	816.3	813.3	$A+Na^+$	PEG^{500} c. [a[	1
	828.0	829.3	$B+Na^+$	PEG^{500} c. [a[	1
	943.8	943.4	$AG+H^+$	PEG^{500} n.c.[a]	1
	977.2	977.4	$BG+H^+$	PEG^{500} n.c.[a]	1
***Am* C_3NMe_3Cl-0.5**	697.9	695.8	DD	C_3NMe_3	1
	811.3	813.0	DD		2
	1099.3	1101.3	DDD		2
	1129.8	1133.3	DBB		2
***Am* C_3CO_2H-0.5**.	496.8	497.5	$D+Na^+$	C_3CO_2H	2
	512.7	513.5	$B+Na^+$		2
	678.5	682.7	$DD+H^+$		1
	685.5	688.7	$CD+Na^+$		1
	700.6	704.7	$DD+Na^+$		1
	732.8	736.7	$BB+Na^+$		!
***Am* PEG_{500}-0.25**	1061.9	1063.6	$CD+H^+$	PEG^{500} c. [a[	1
	1093.1	1097.6	$DD+H^+$	PEG^{500} c. [a[	1
	1238.3	1238.7	$CDG+Na^+$	PEG^{500} c.[a[	1
		1239.7	$CCG+Na^+$	PEG^{500} n.c.[a]	1

Table S2: MALDI-ToF analysis of functionalised hydrlysable tannins. For letter codes of identified monomeric tannin building blocks refer to Figure S11, for functionals to Figure 3.

Functionalised tannin	Observed mass peak [Da]	Calculated mass [Da]	Assignment		
			base strucure	funtional	# functional
**Ta-01 C_3NMe_3Cl-0.5**	441.5		L8+1Na+	C_3NMe_3	3
**Ta-01 C_3CO_2H-0.5**	999.2	995.8	$L4+H^+$	C_3CO_2H	2
	1609.3	1604.2	$L8+H^+$		2
**Ta-04 C_3NMe_3Cl-0.5**	394.1	391.4	$Q4+Na^+$	C_3NMe_3	3
	458.6	461.5	$Q1+Na^+$		1
	679.4 o 684.9	681.1	$Q6+2Na^+$		1
**Ta-04 C_3CO_2H-0.5**	446.0	448.4	$Q1+H^+$	C_3CO_2H	1
	622.7	624.8	$Q9+3H^+$		1
	927.2	926.7	$Q4+Na^+$		1
	1079.9	1078.8	$Q5+Na^+$		1
	1232.6	1230.9	$Q6+Na^+$		1
	1385.1	1383.0	Q7+Na+		1

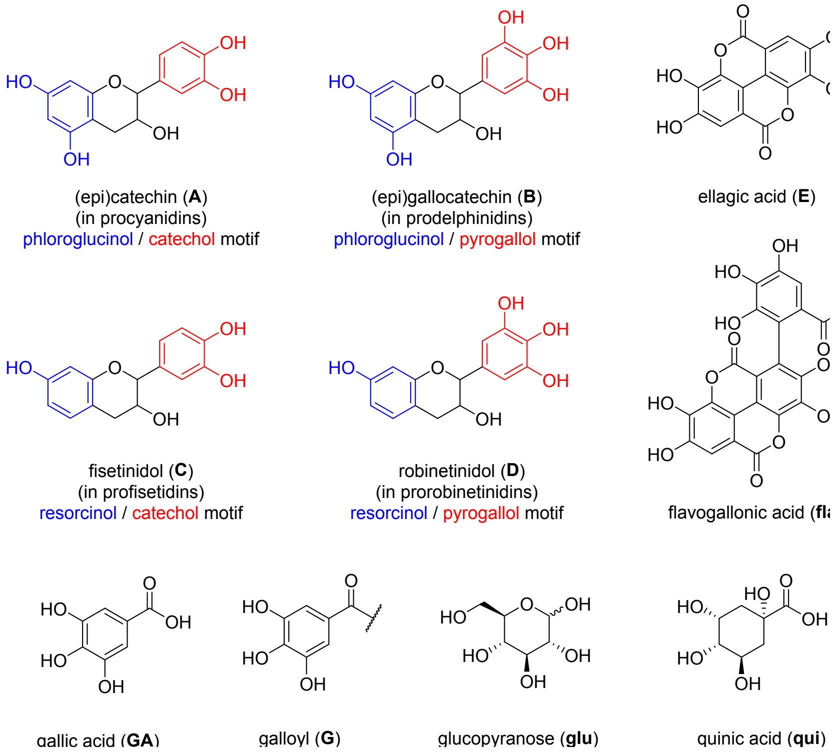

Figure S11: Structural elements used for discussing the various analysis data.
